# Supplementary material for: Brain hothubs and dark functional networks: correlation analysis between amplitude and connectivity for Broca’s aphasia
Source: PeerJ. 2020 Oct 1;8:e10057. doi: 10.7717/peerj.10057 (PMC7533062; doi:10.7717/peerj.10057)
Supplement: File S1 — The 40 black-and-white outline drawings from the 100 words of the Kent-Rosanoff word association test. [file peerj-08-10057-s001.pdf]

Catalogue

baby	2
bath	3
bed	4
blossom	5
boy	6
bread	7
butterfly	8
cabbage	9
carpet	10
chair	11
child	12
doctor	13
eagle	14
earth	15
foot	16
fruit	17
girl	18
hammer	19
hand	20
house	21
king	22
lamp	23
lion	24
man	25
moon	26
mountain	27
needle	28
priest	29
scissors	30
sheep	31
sleep	32
soldier	33
square	34
table	35
thief	36
tobacco	37
whistle	38
window	39
woman	40
working	41

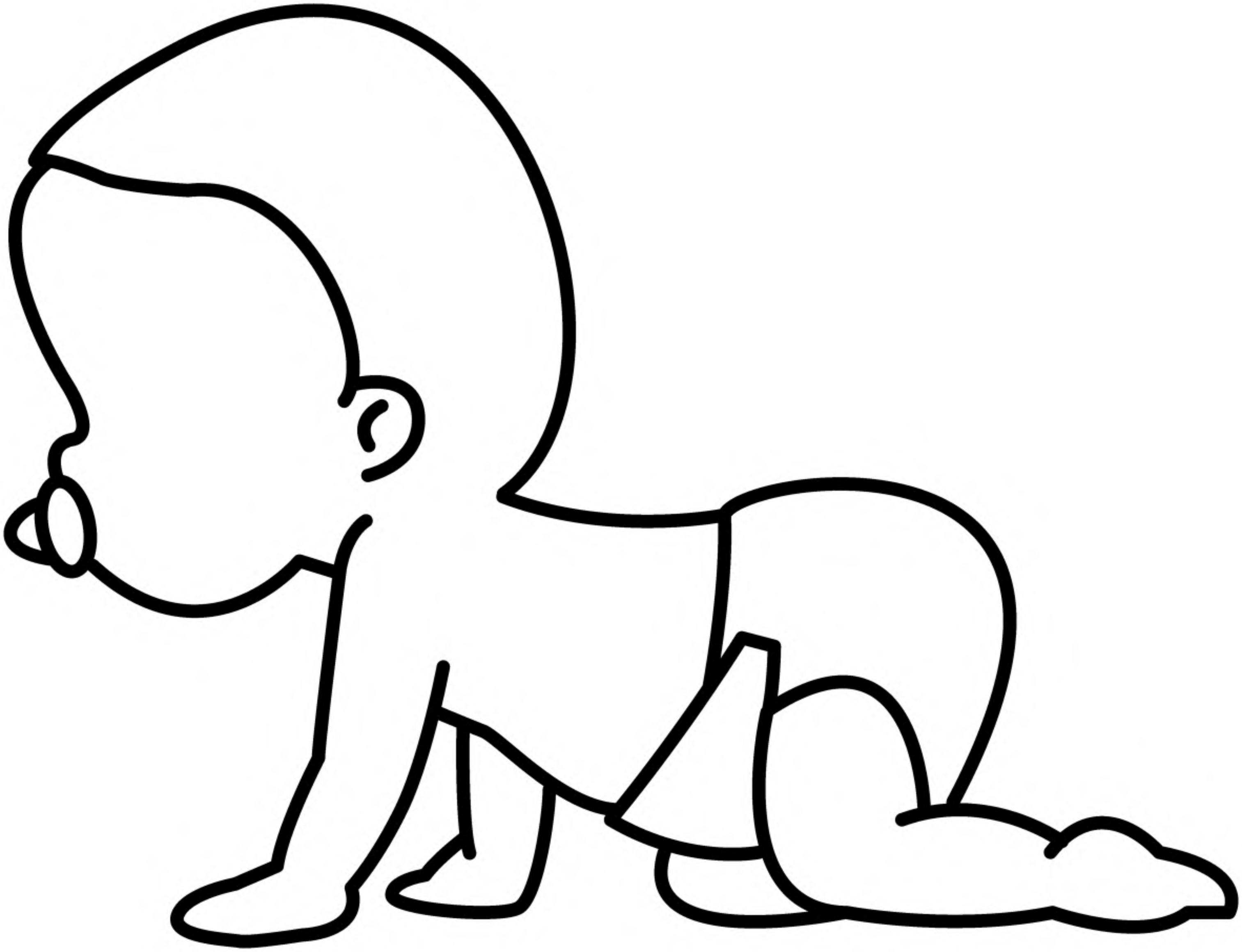

baby

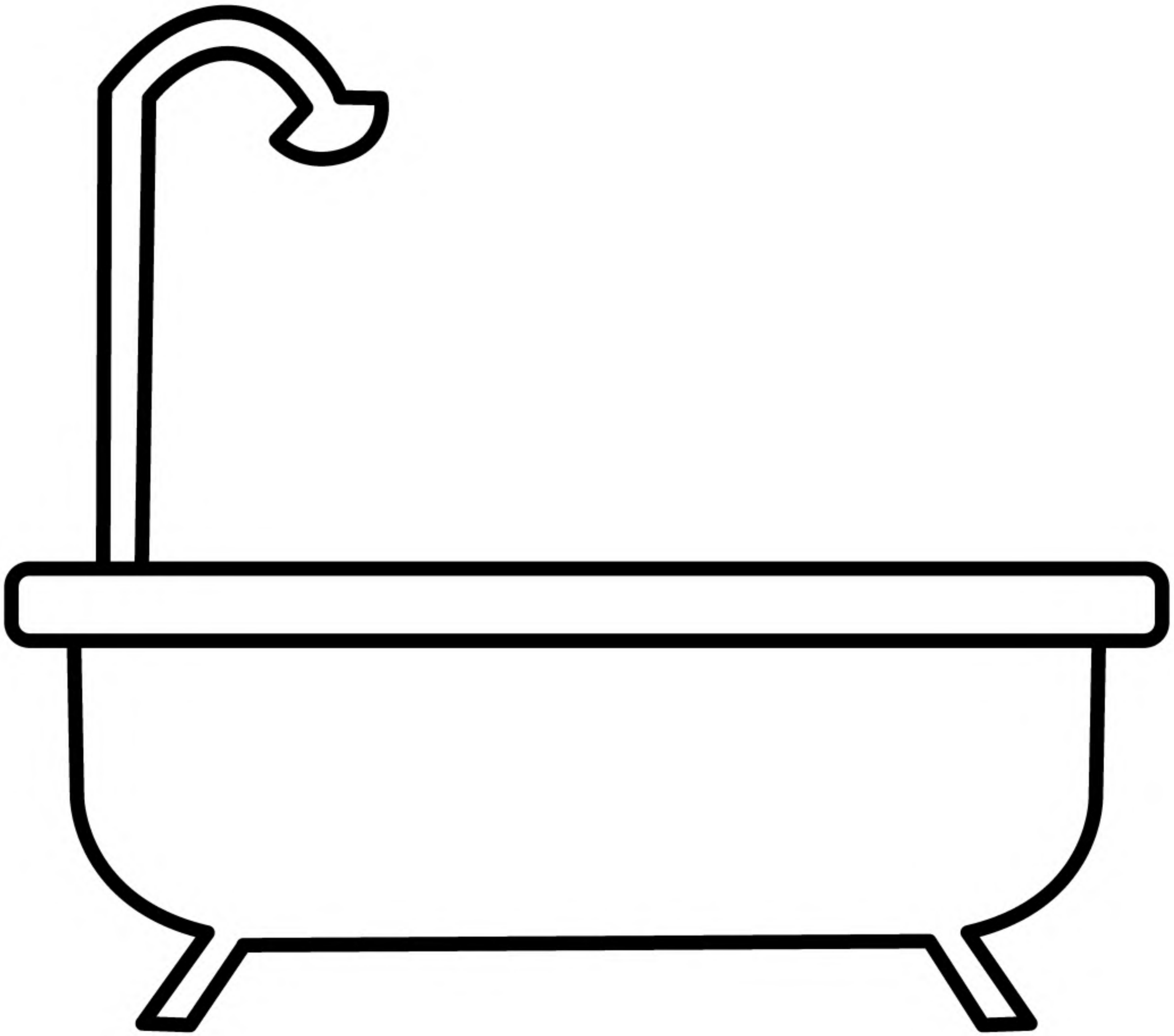

bath

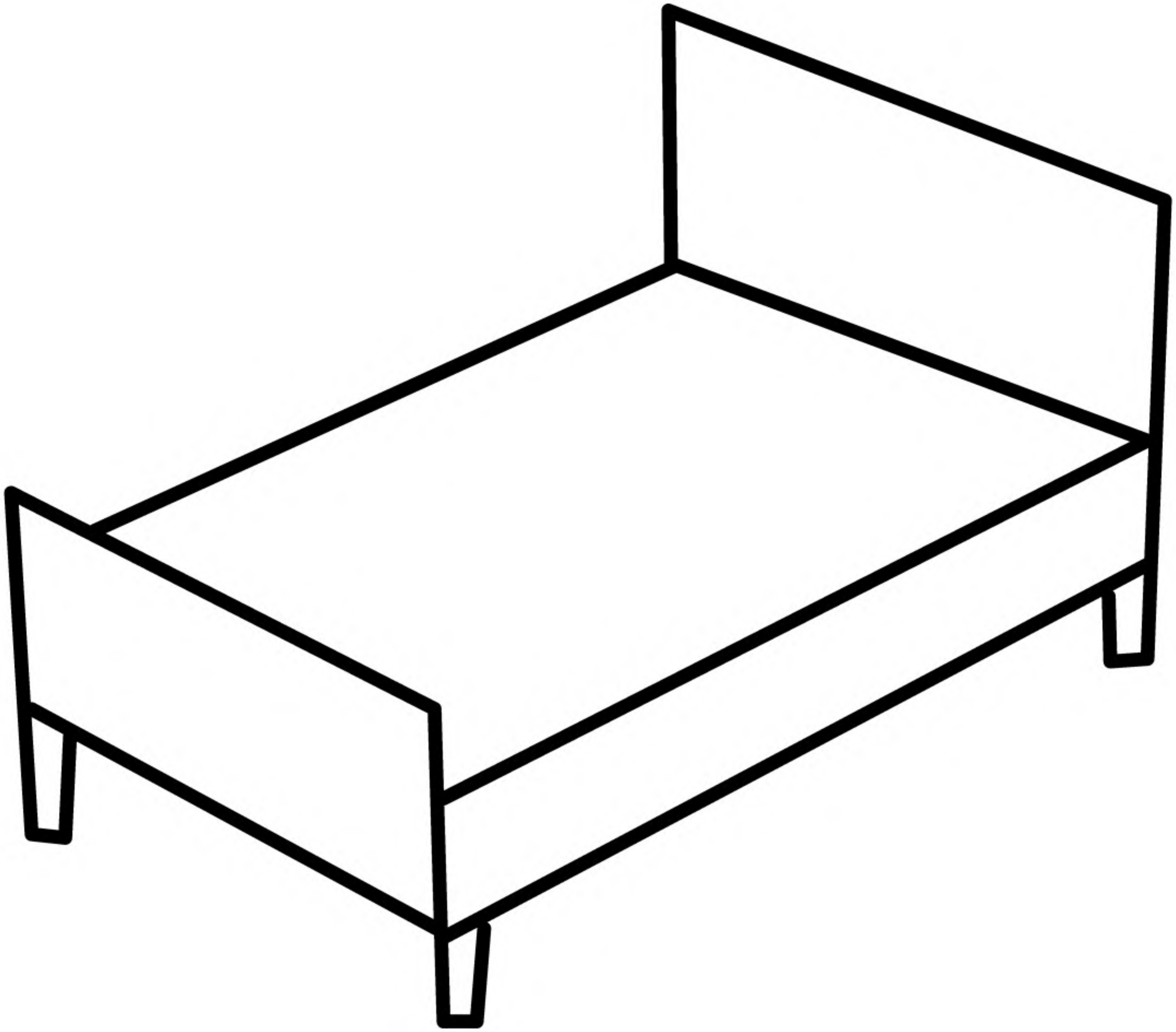

bed

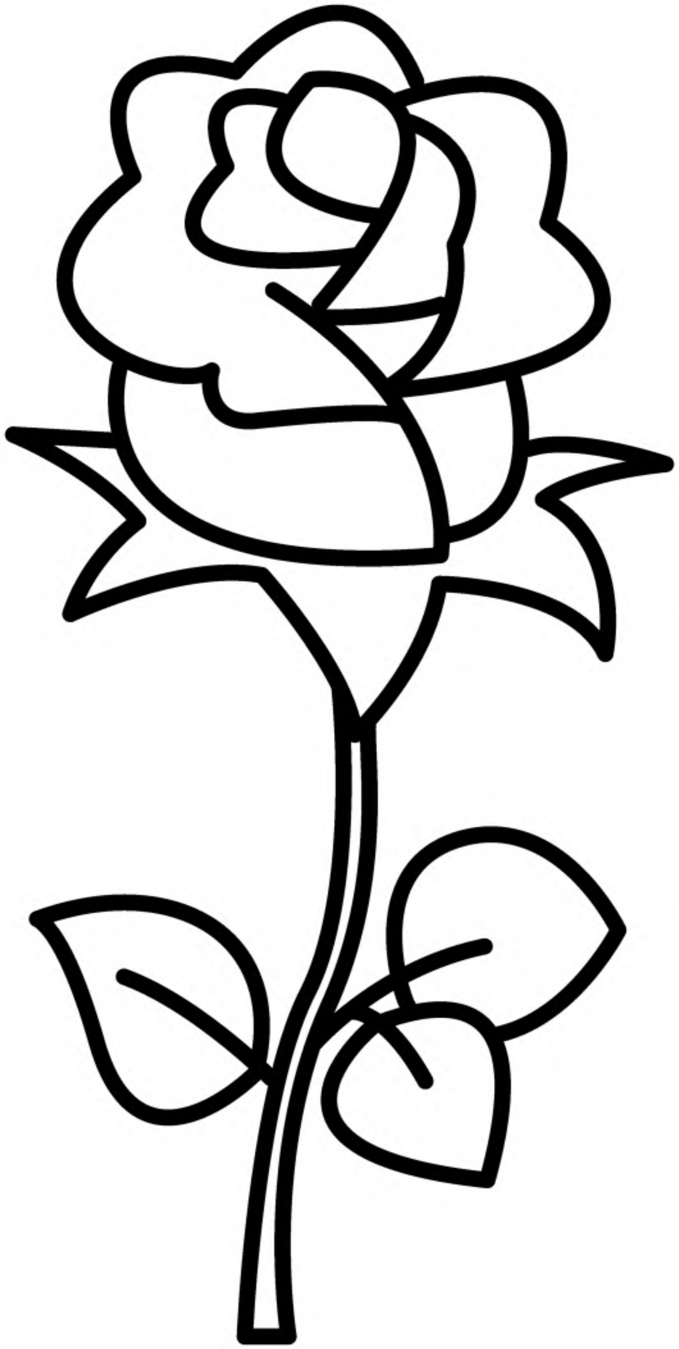

blossom

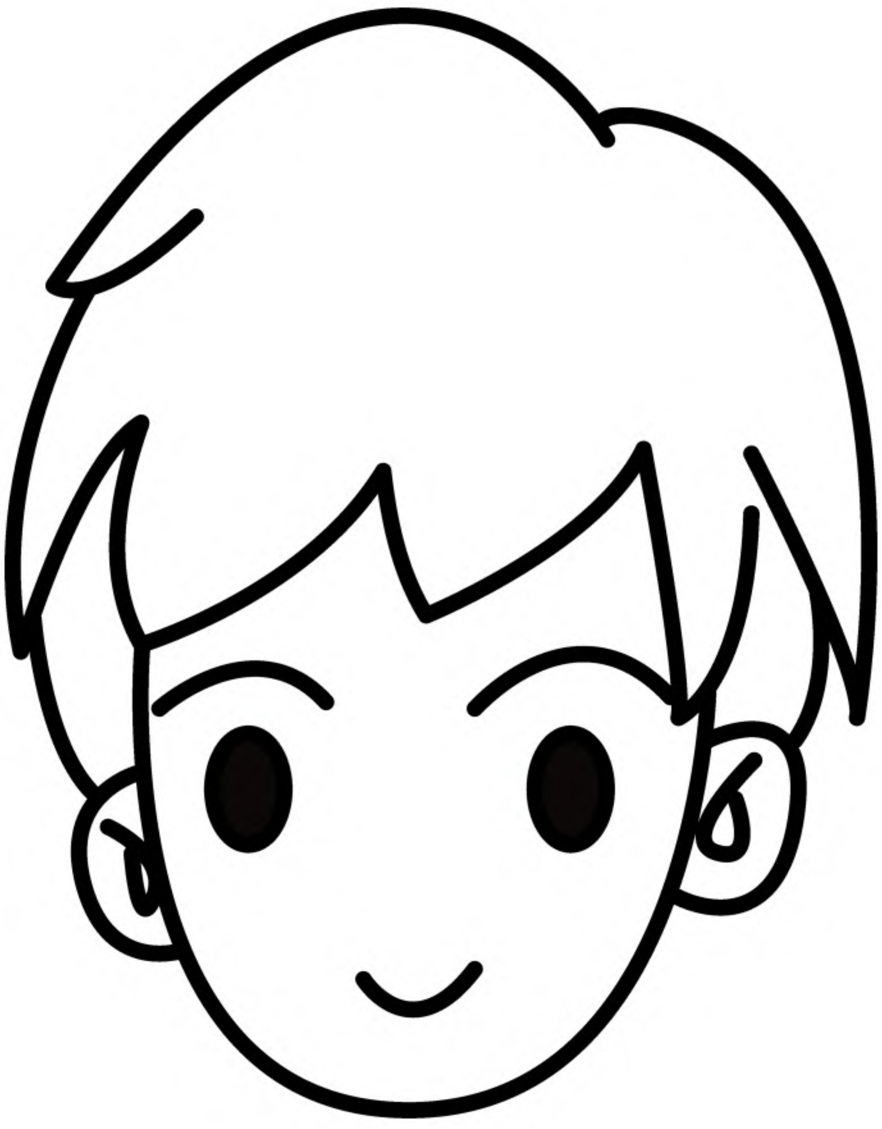

boy

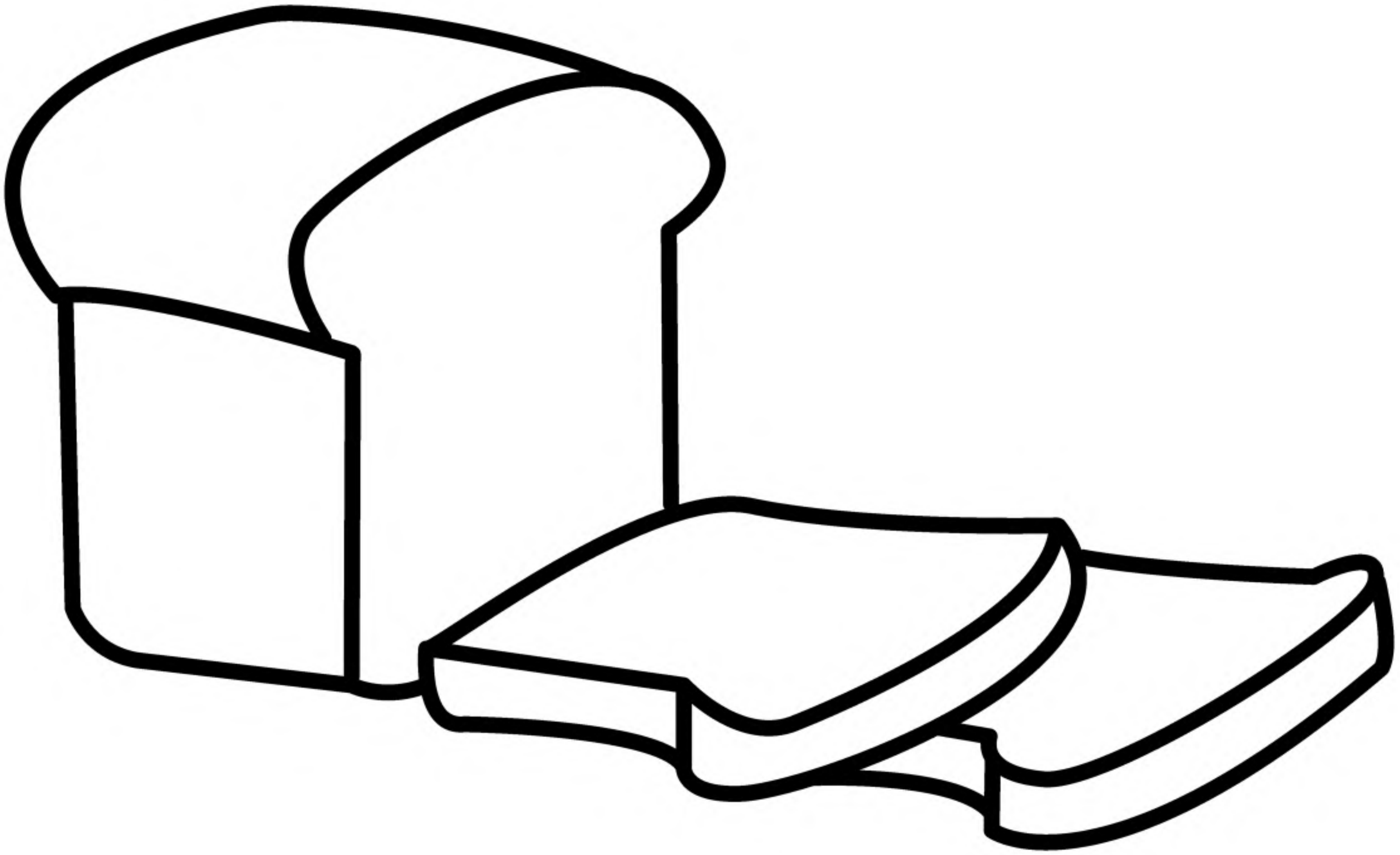

bread

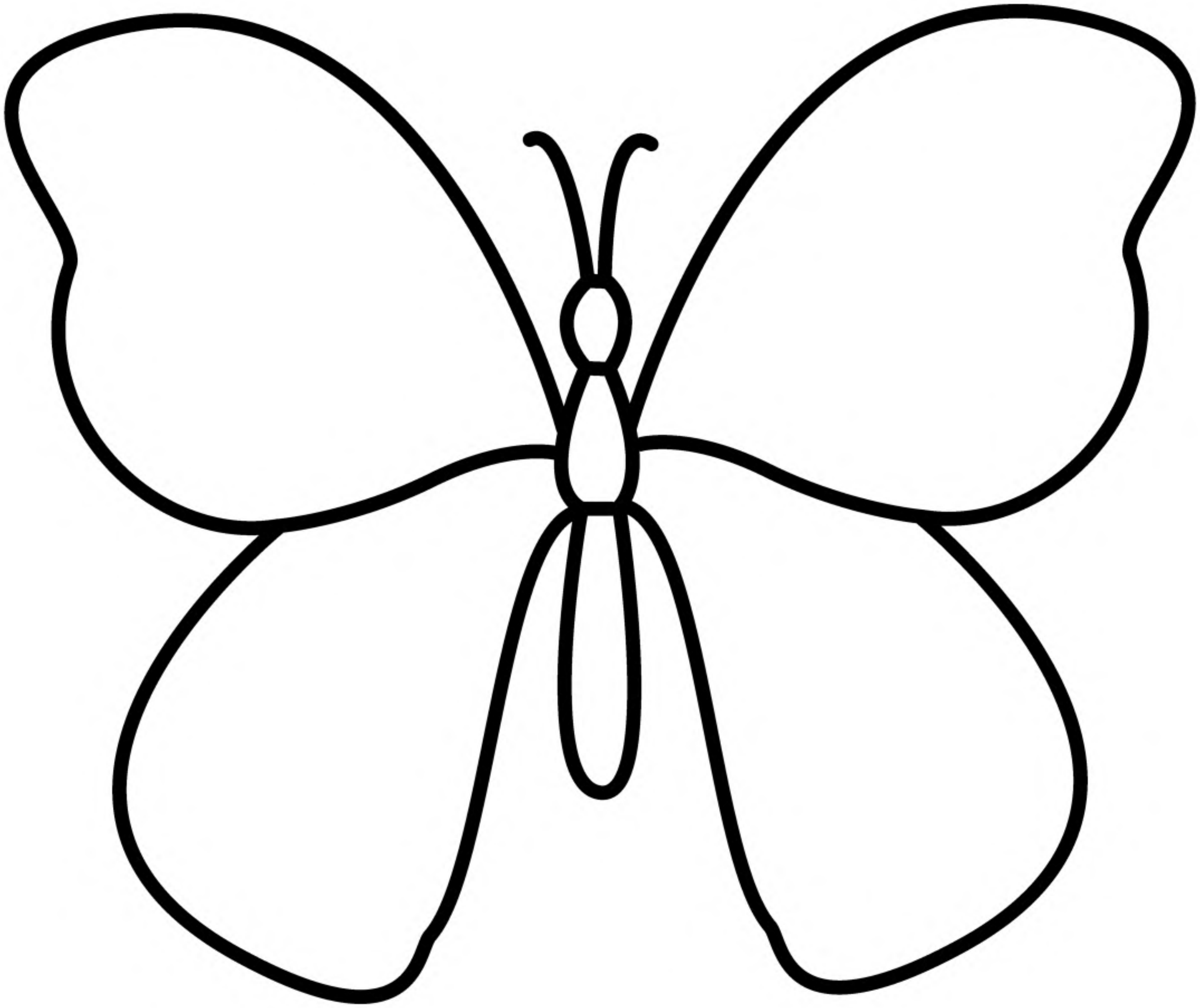

butterfly

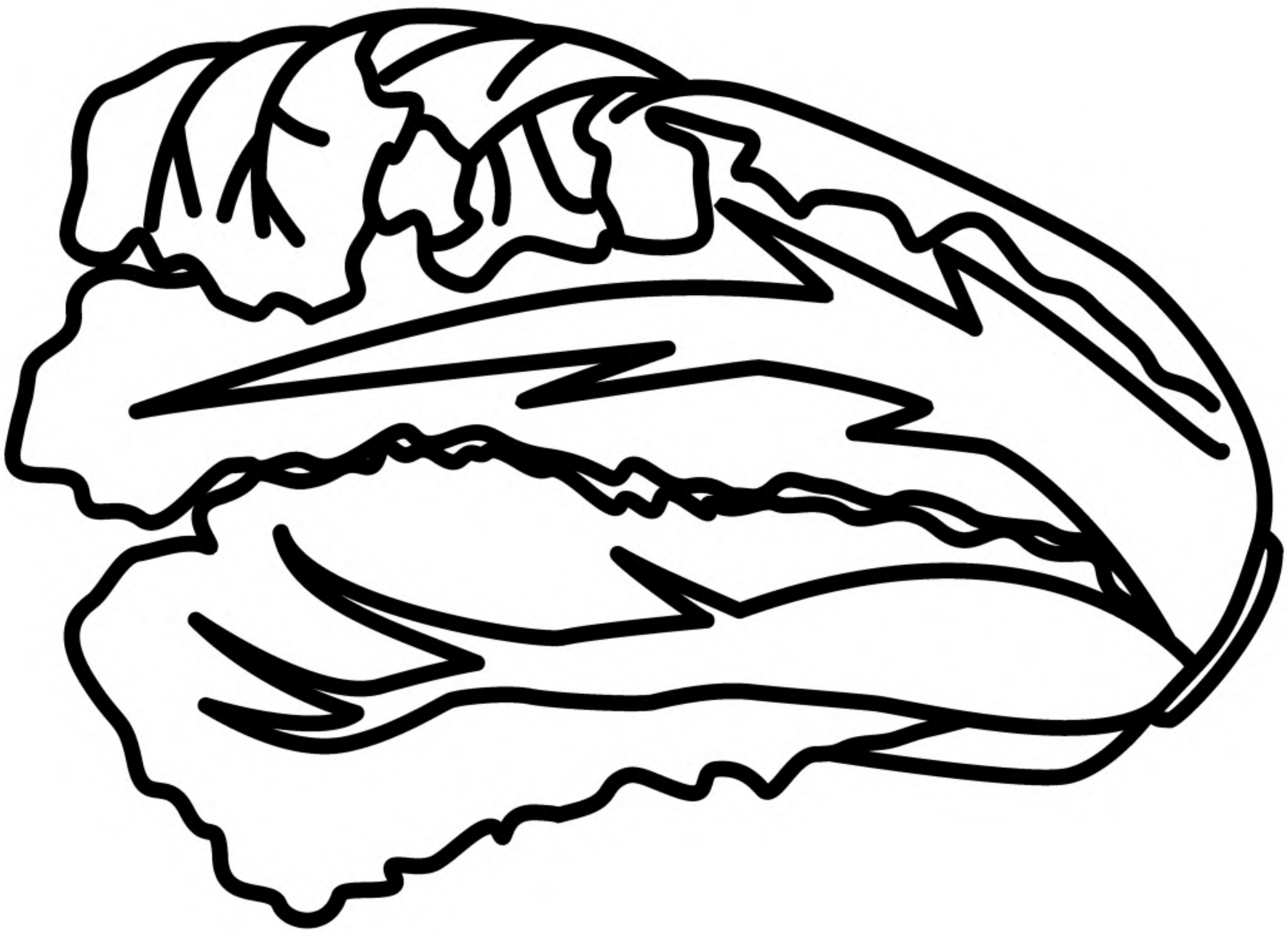

cabbage

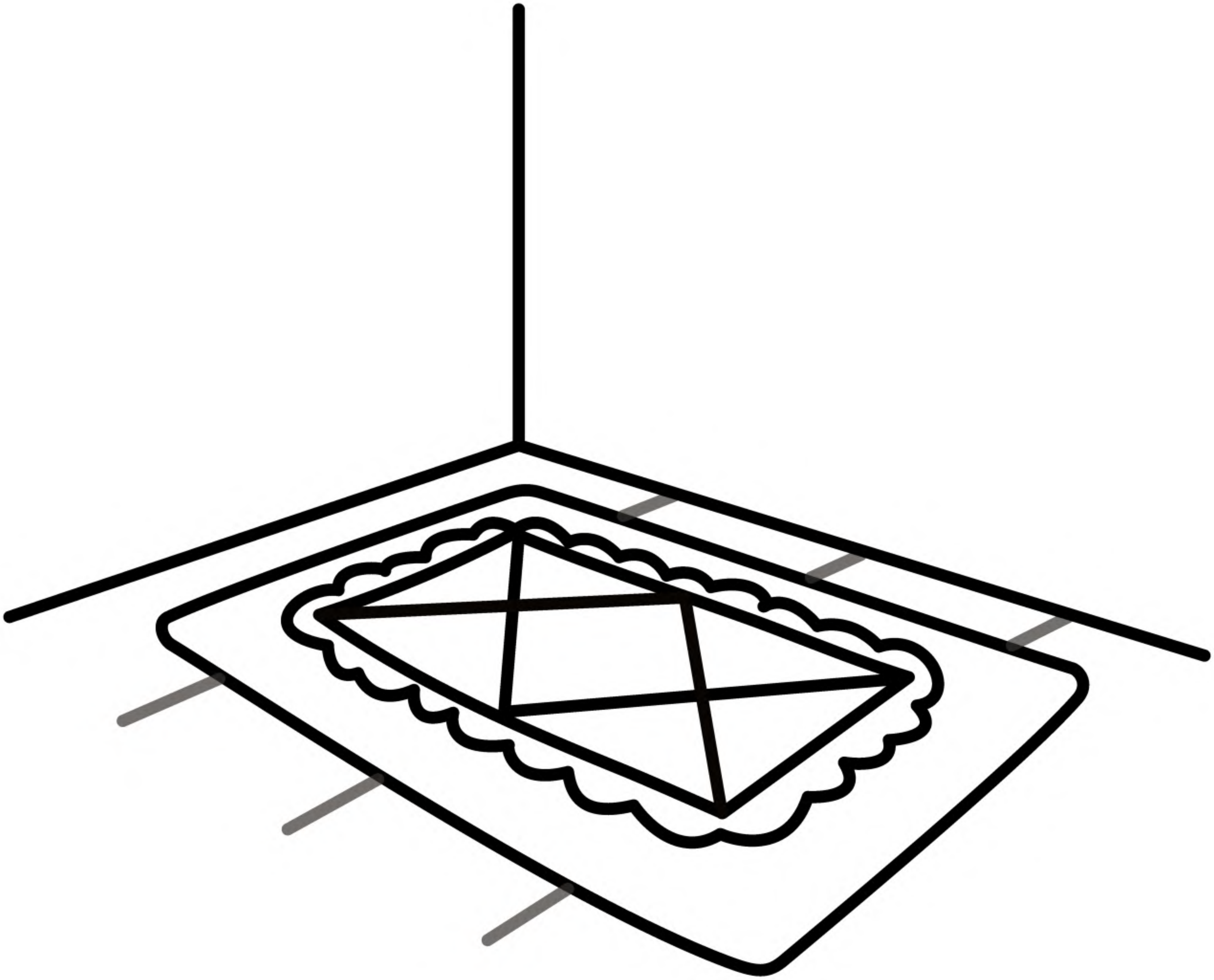

carpet

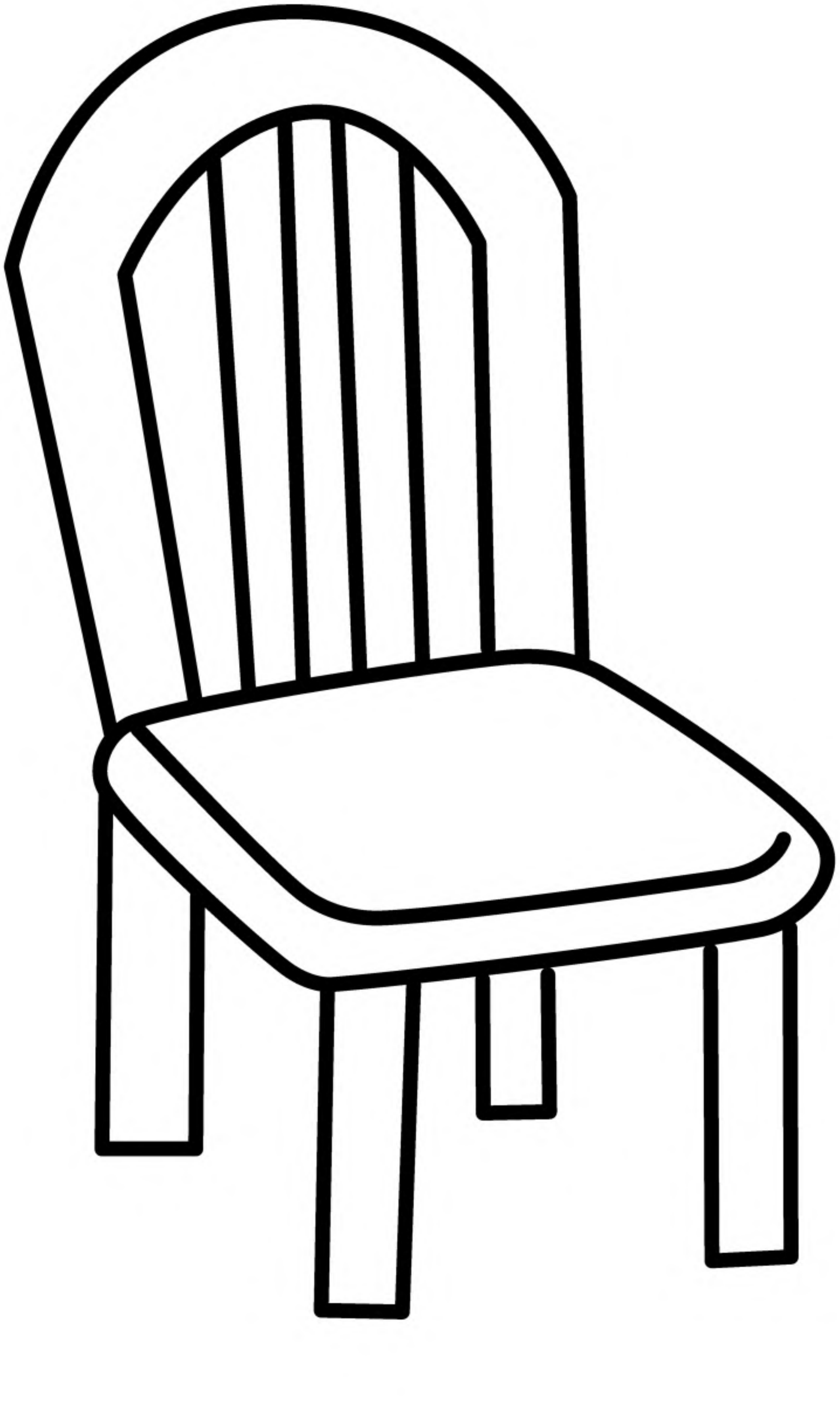

chair

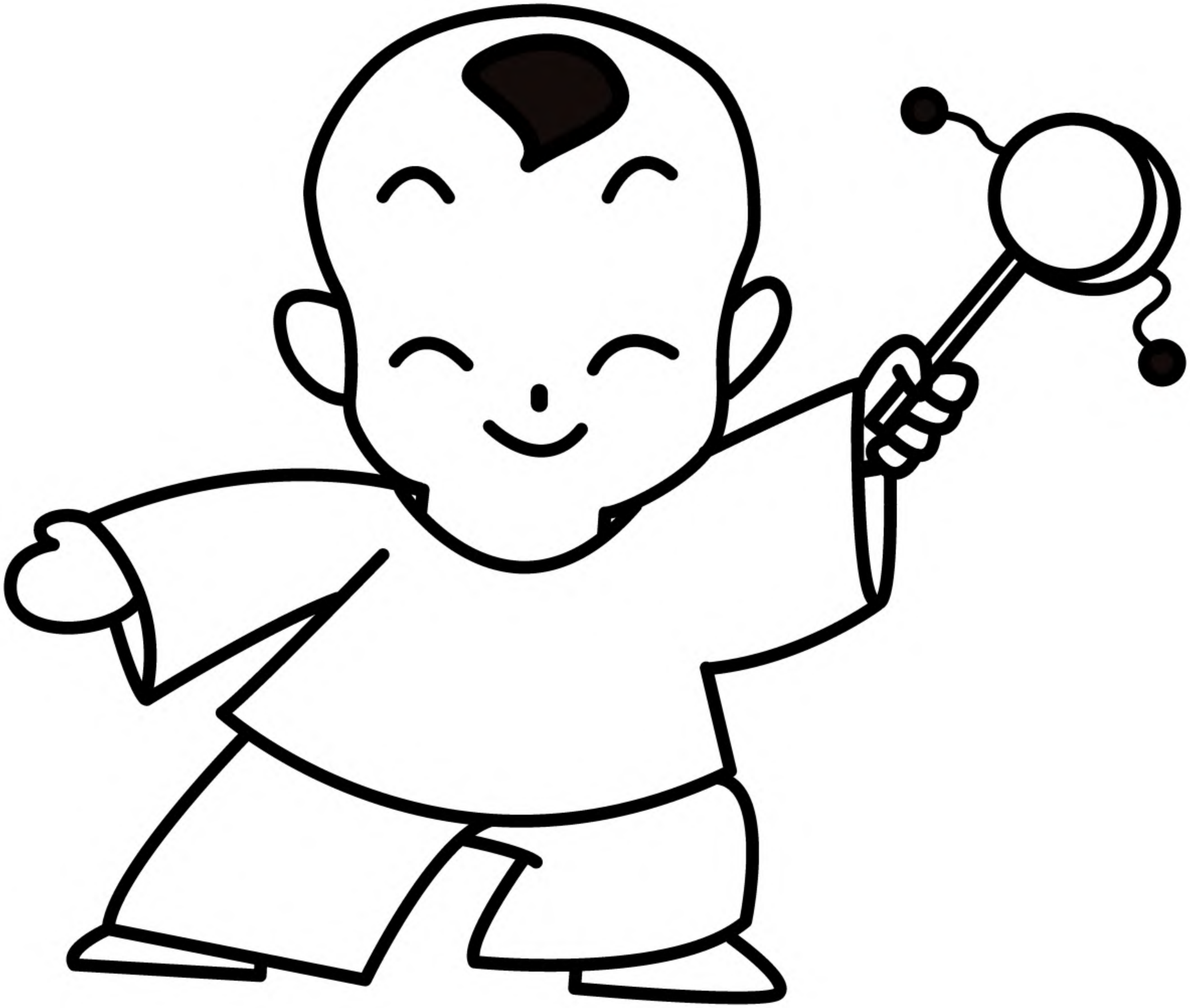

child

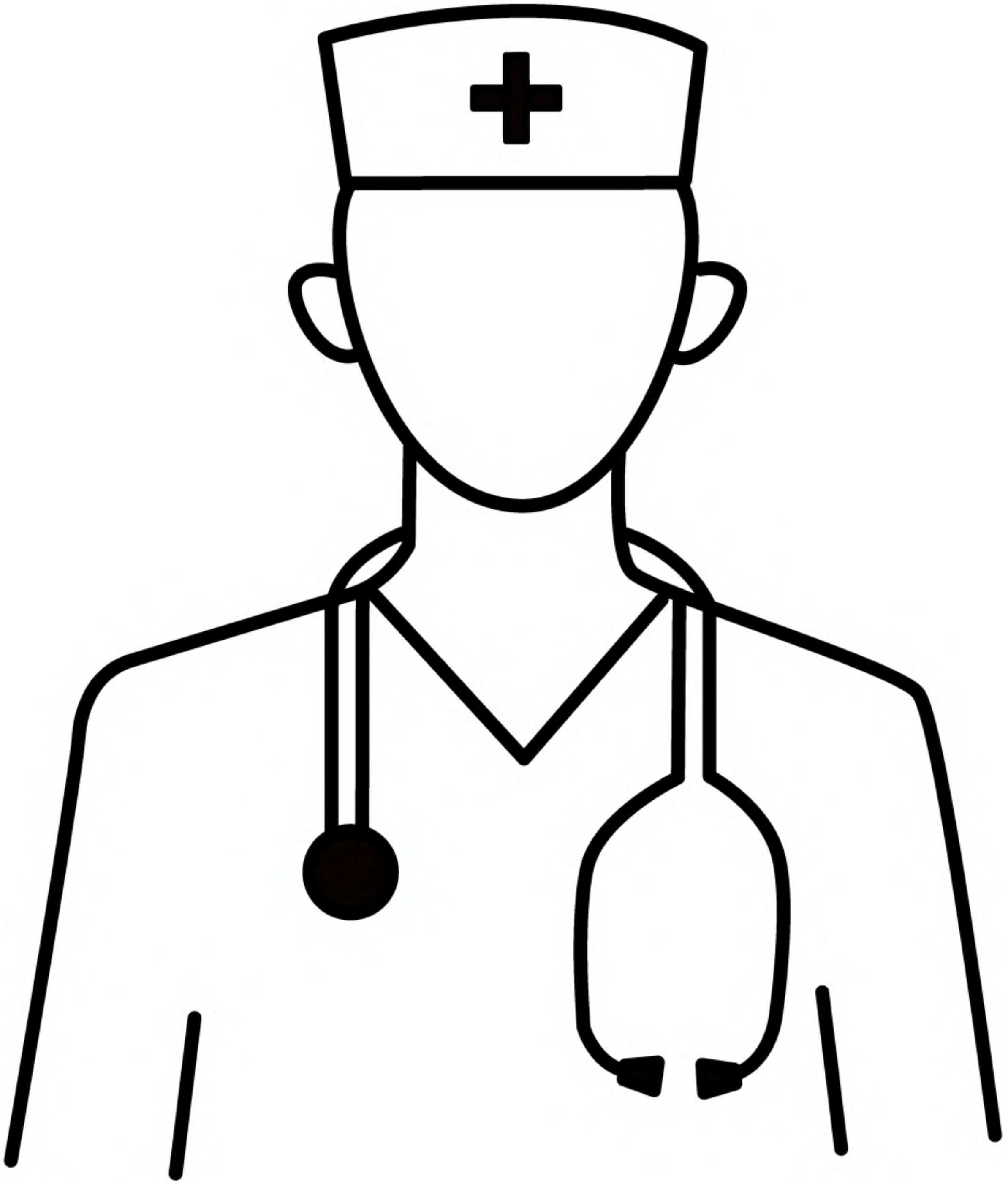

doctor

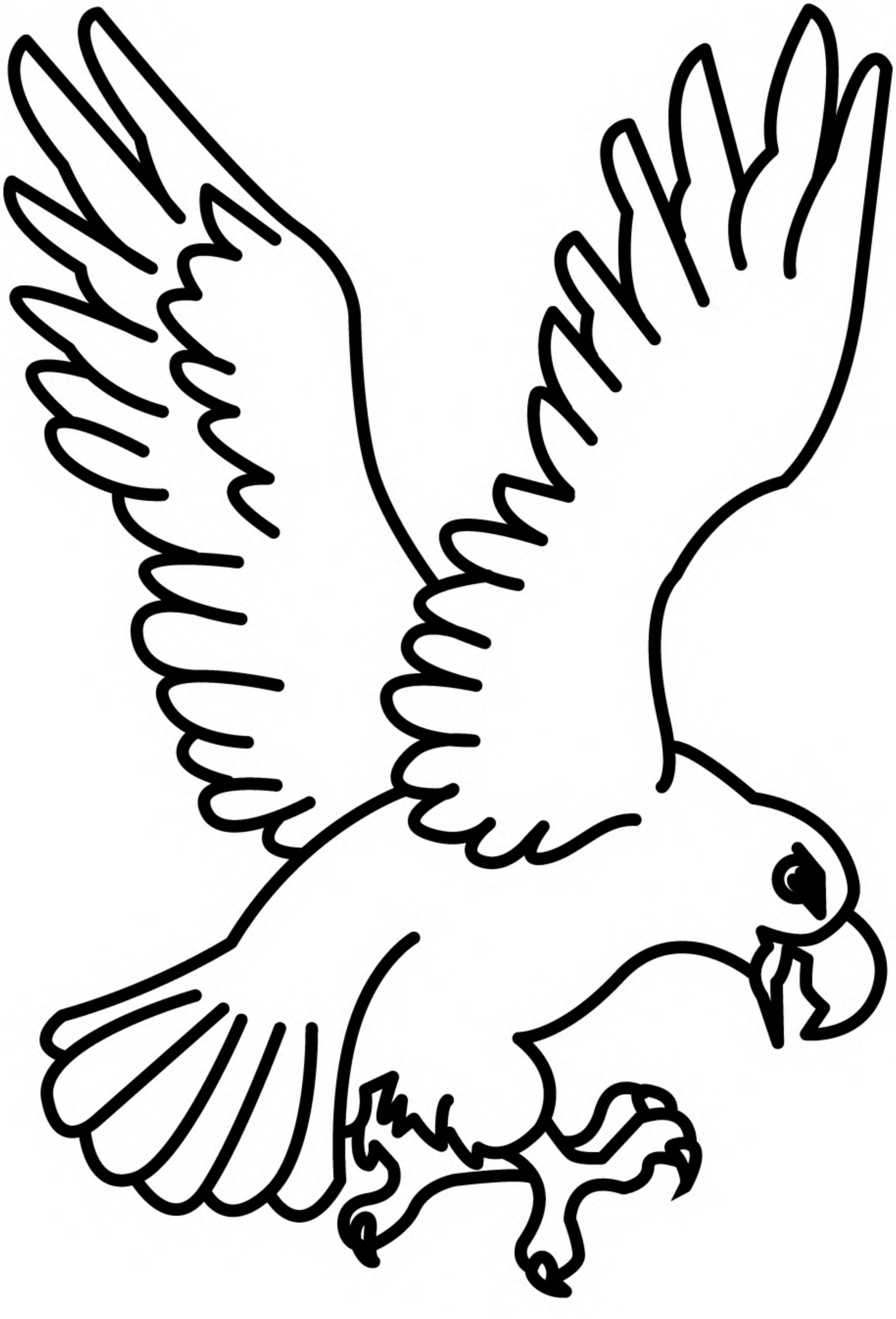

eagle

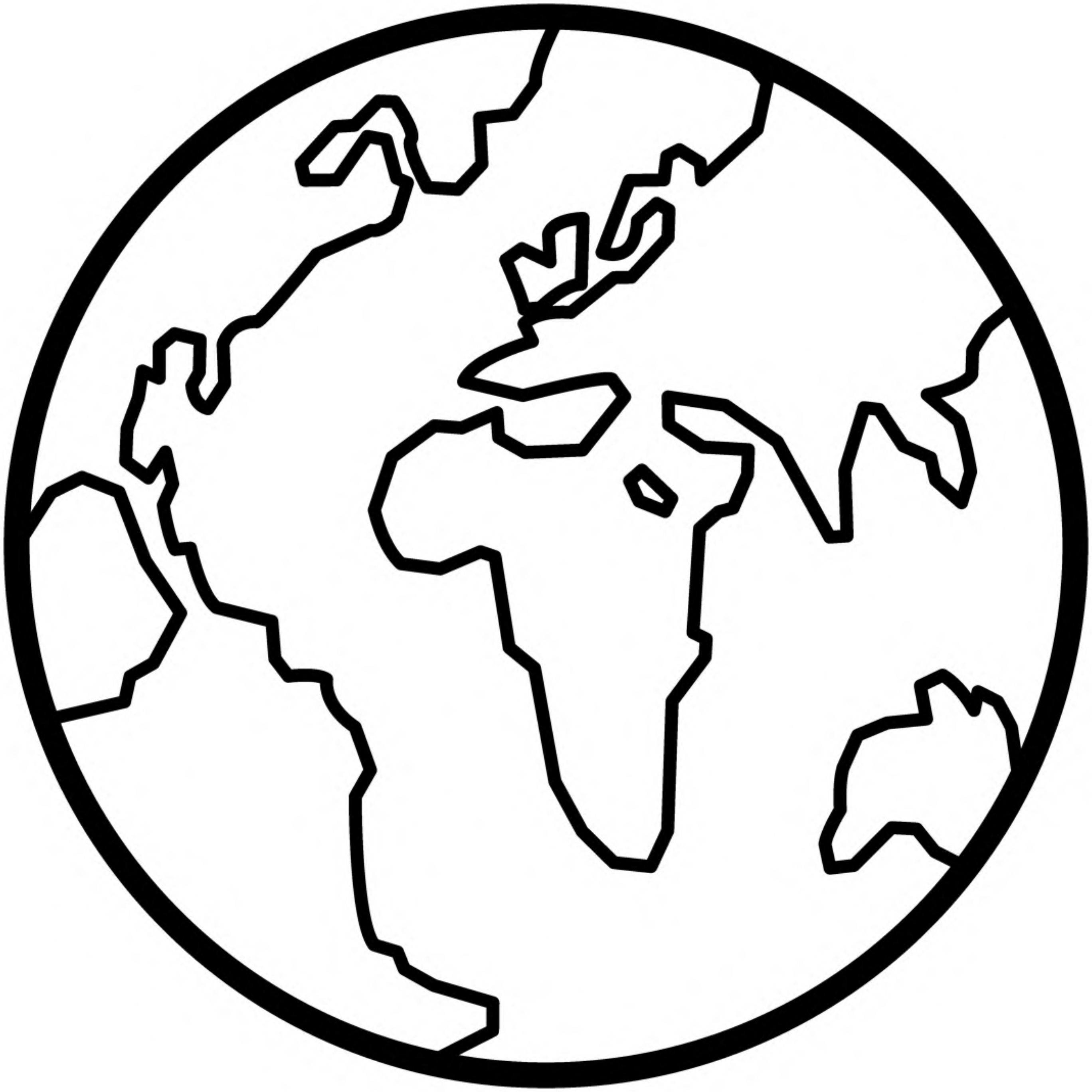

earth

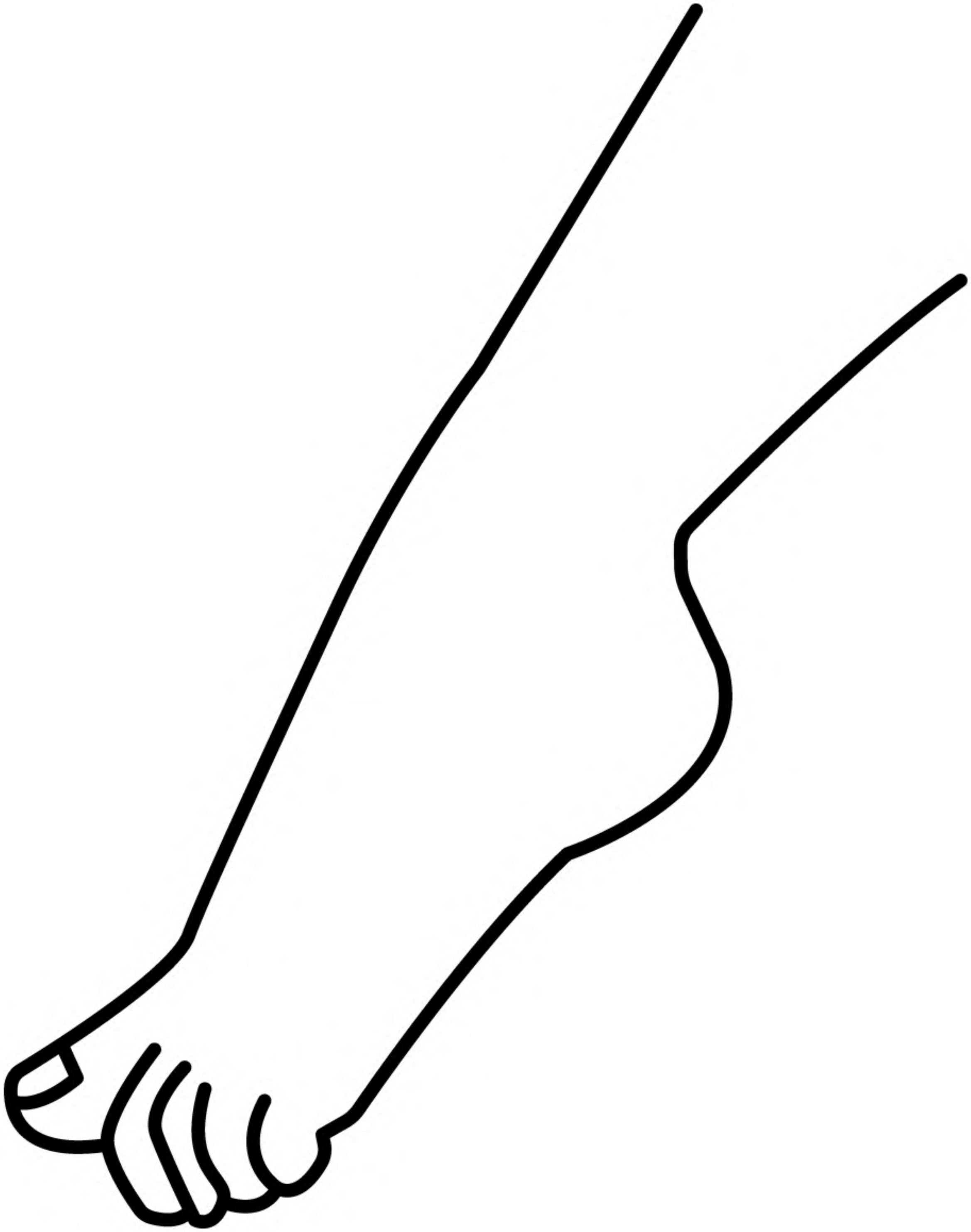

foot

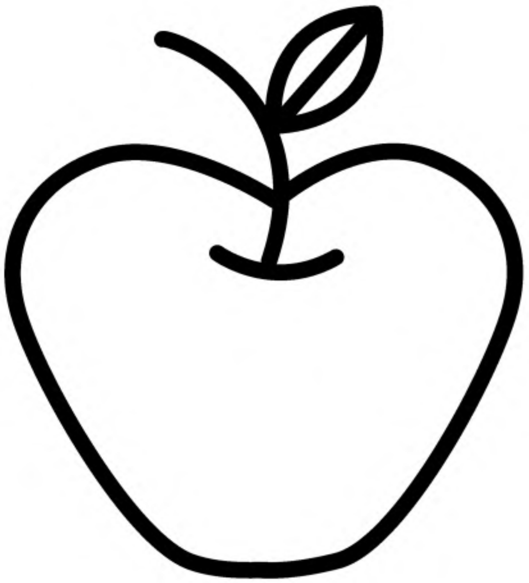

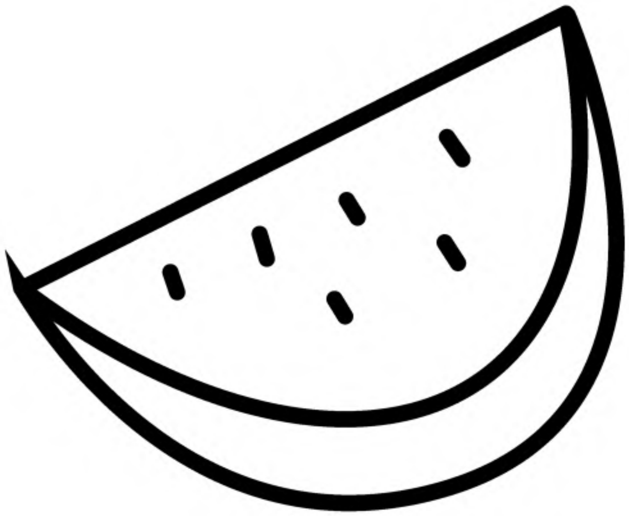

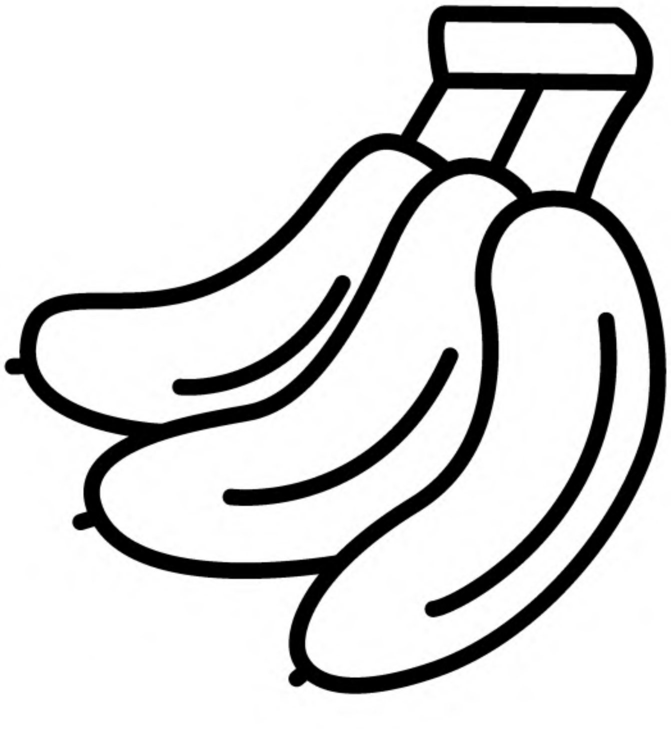

fruit

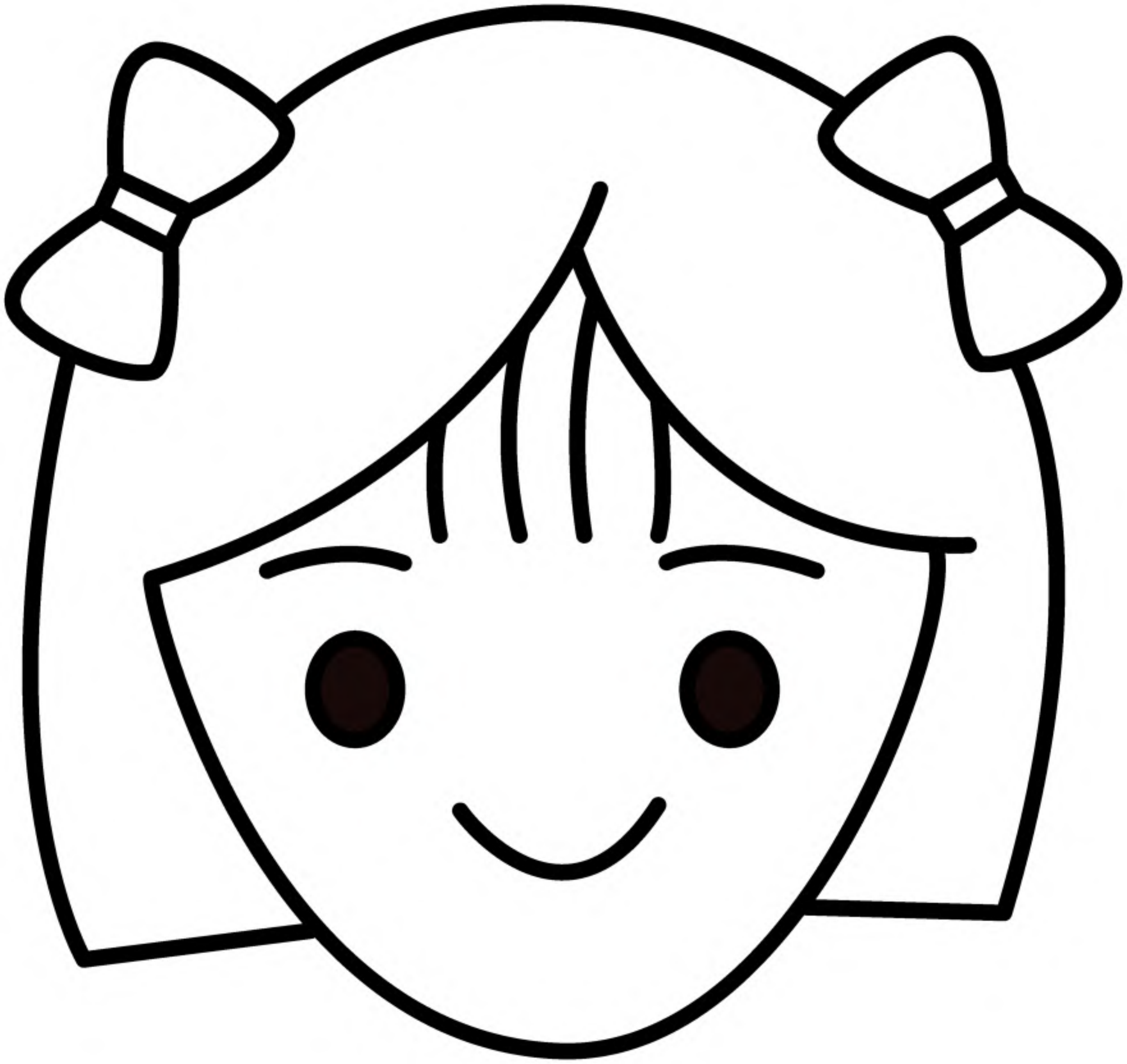

girl

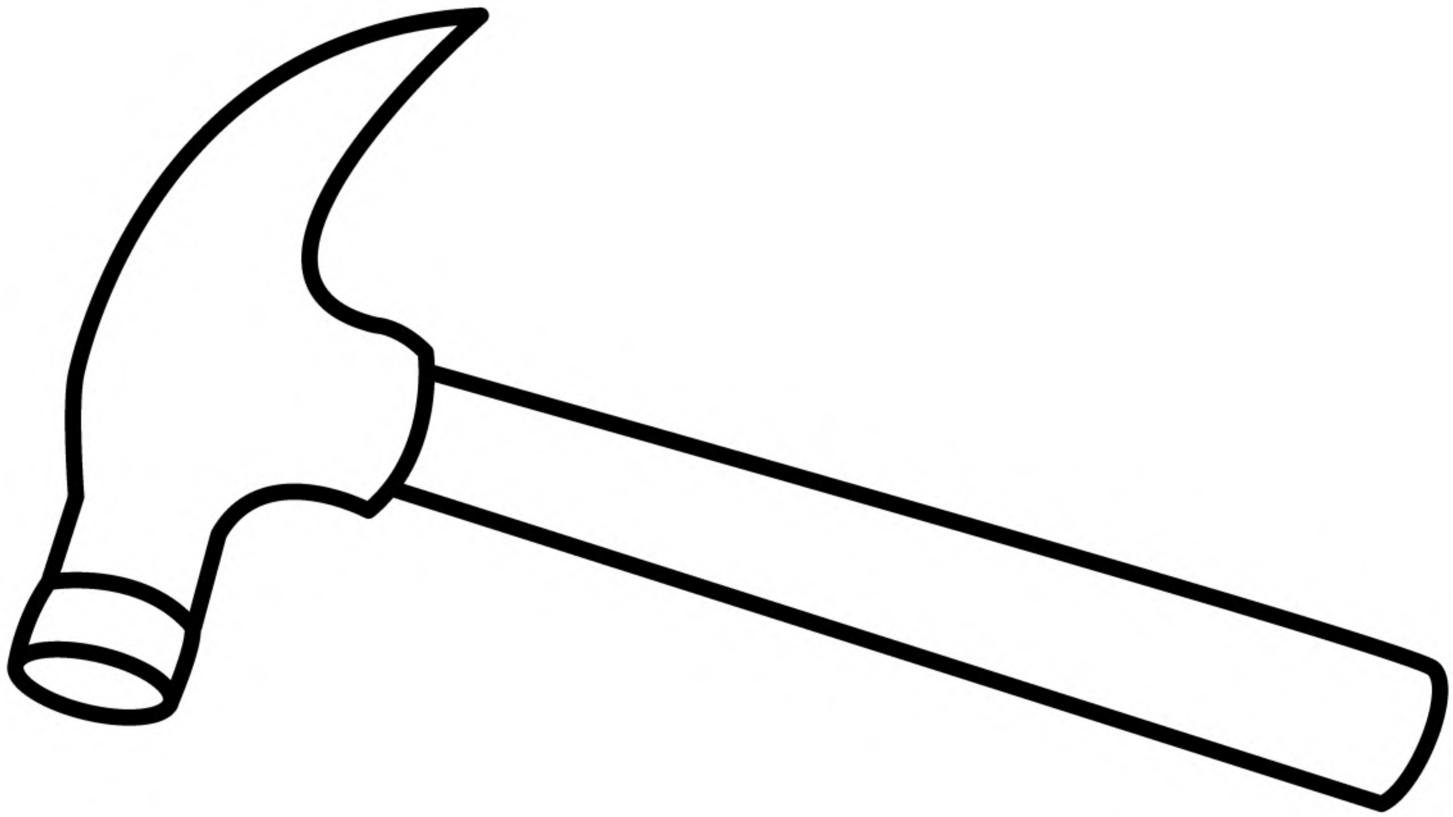

hammer

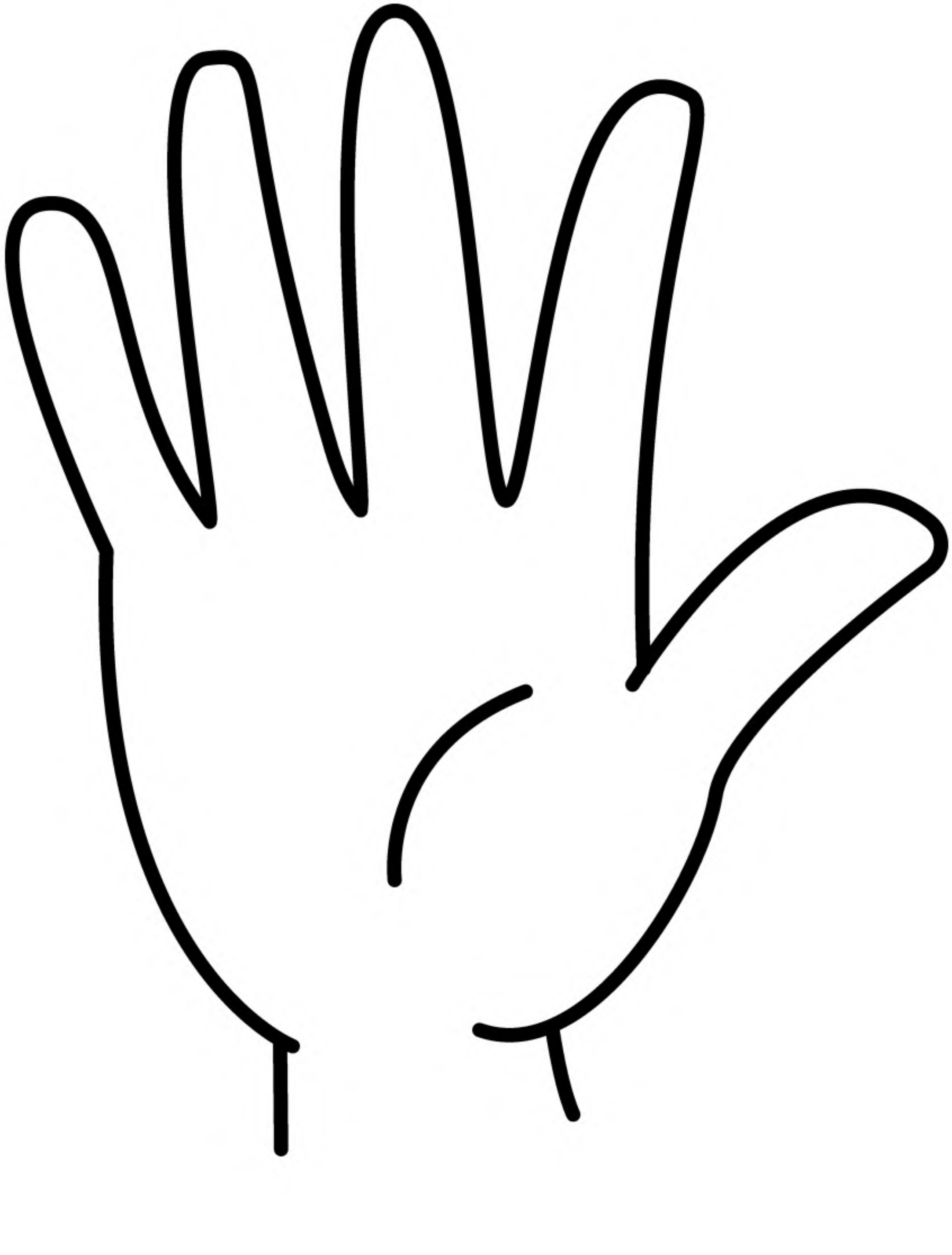

hand

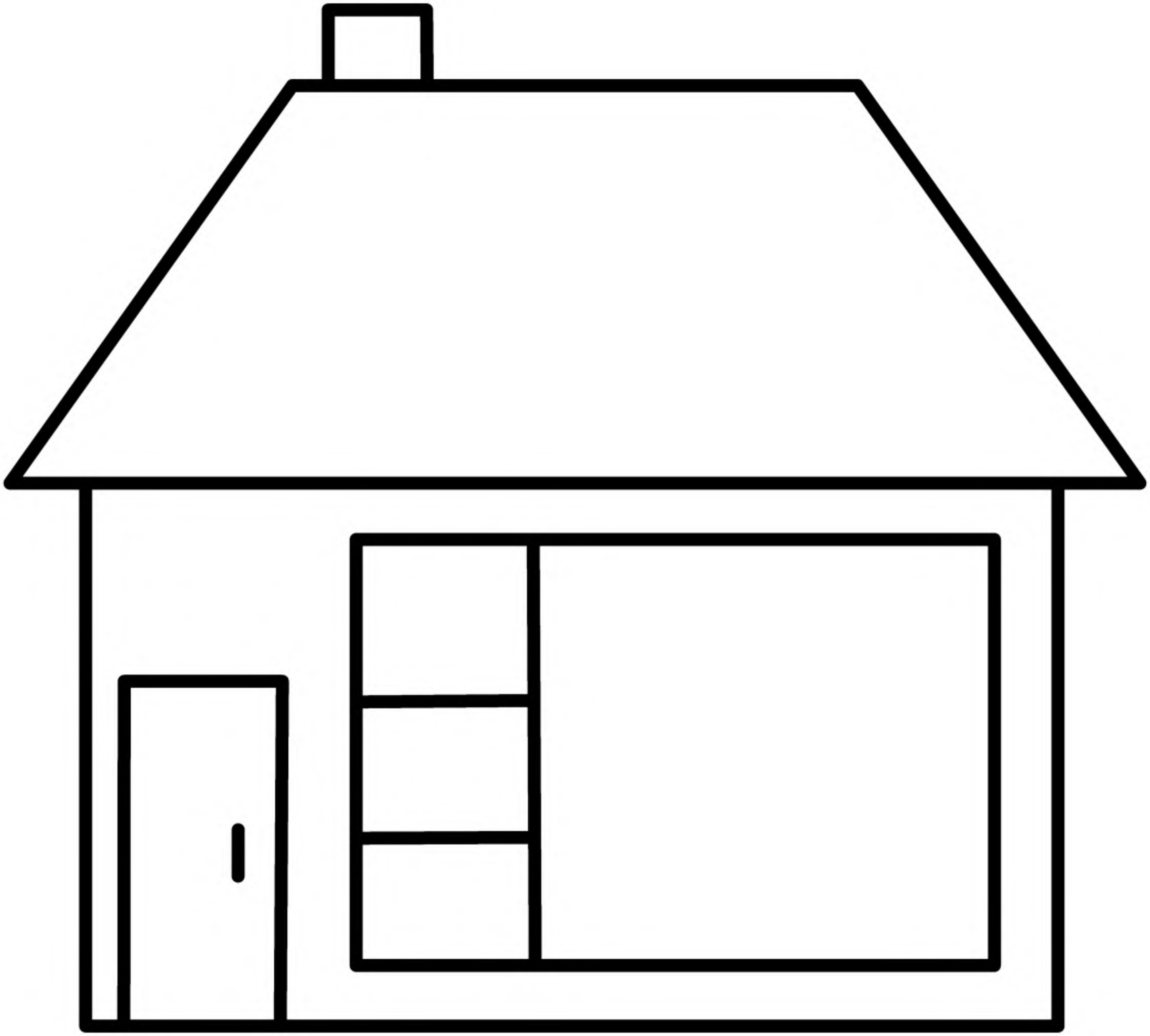

house

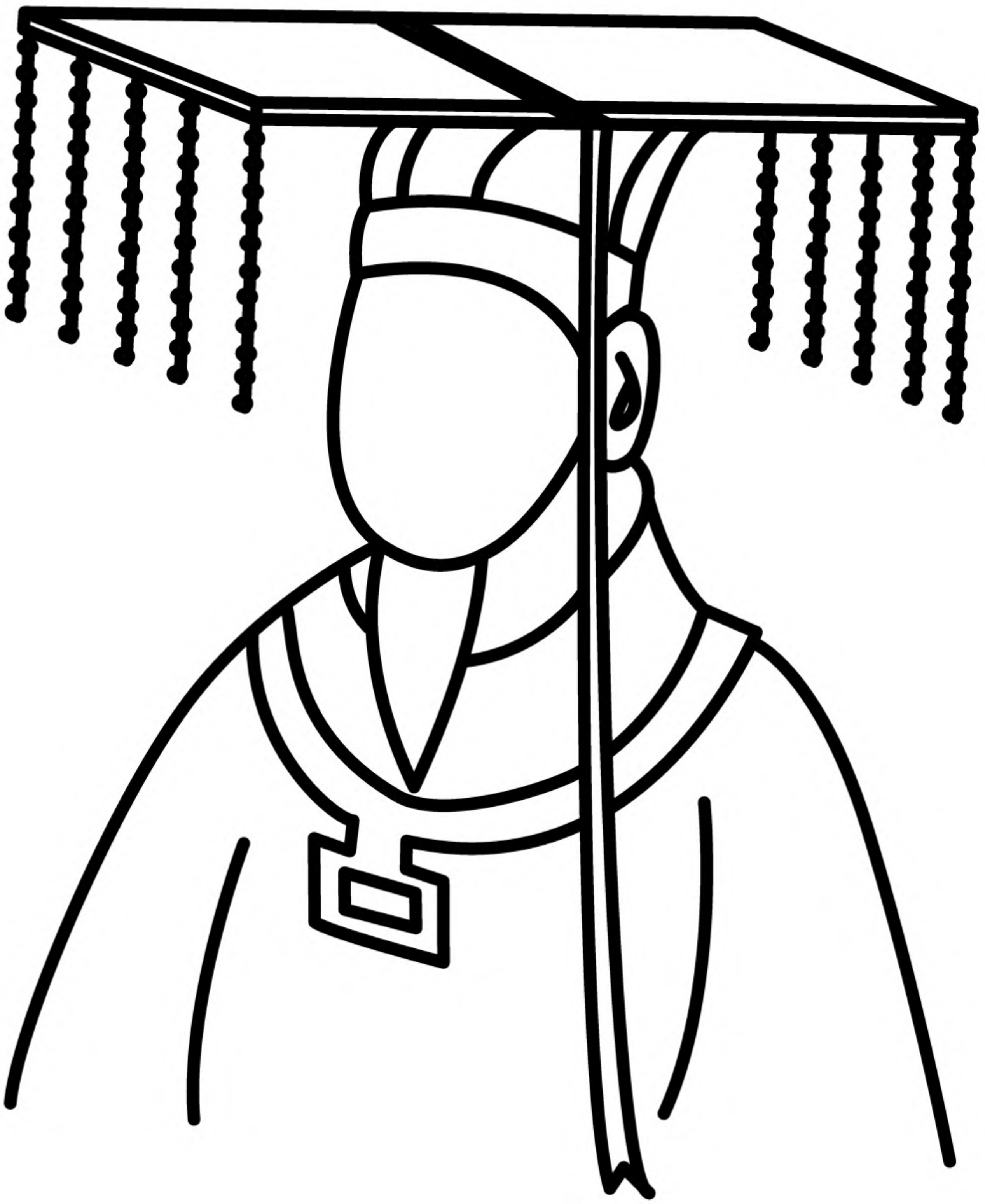

king

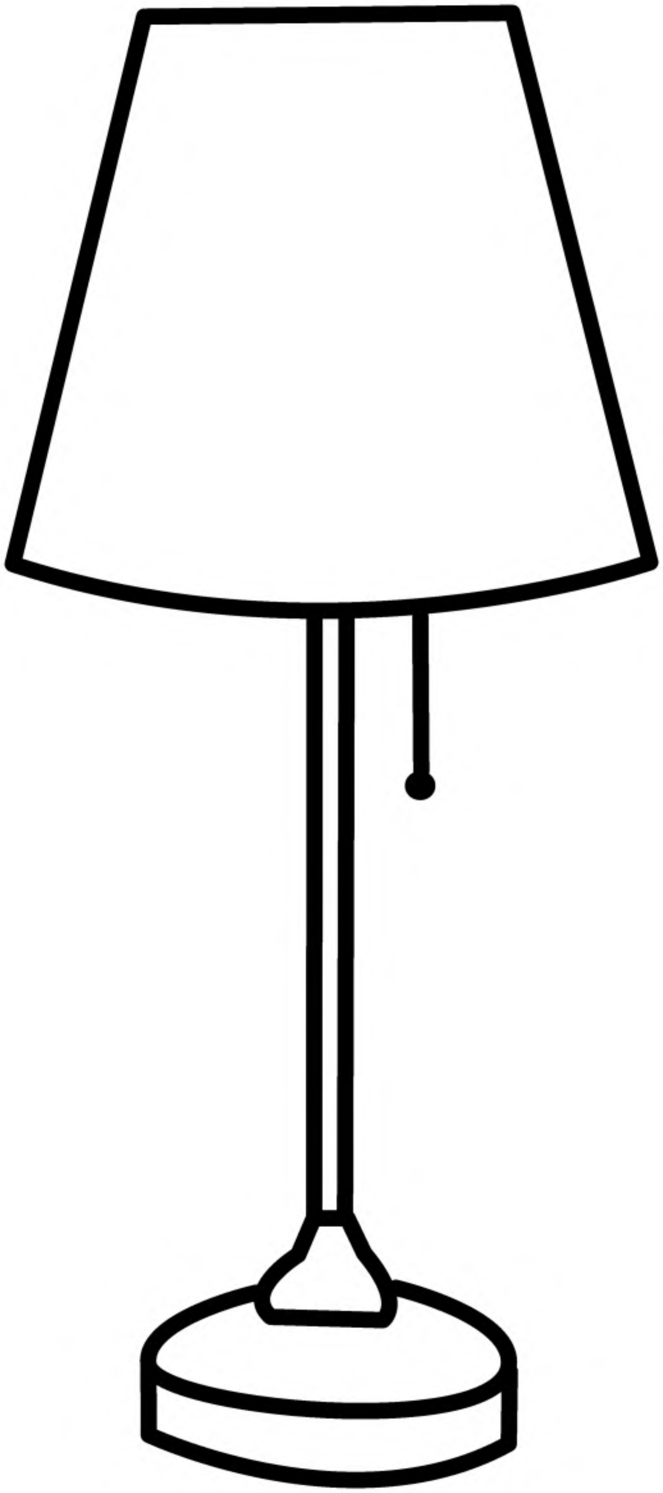

lamp

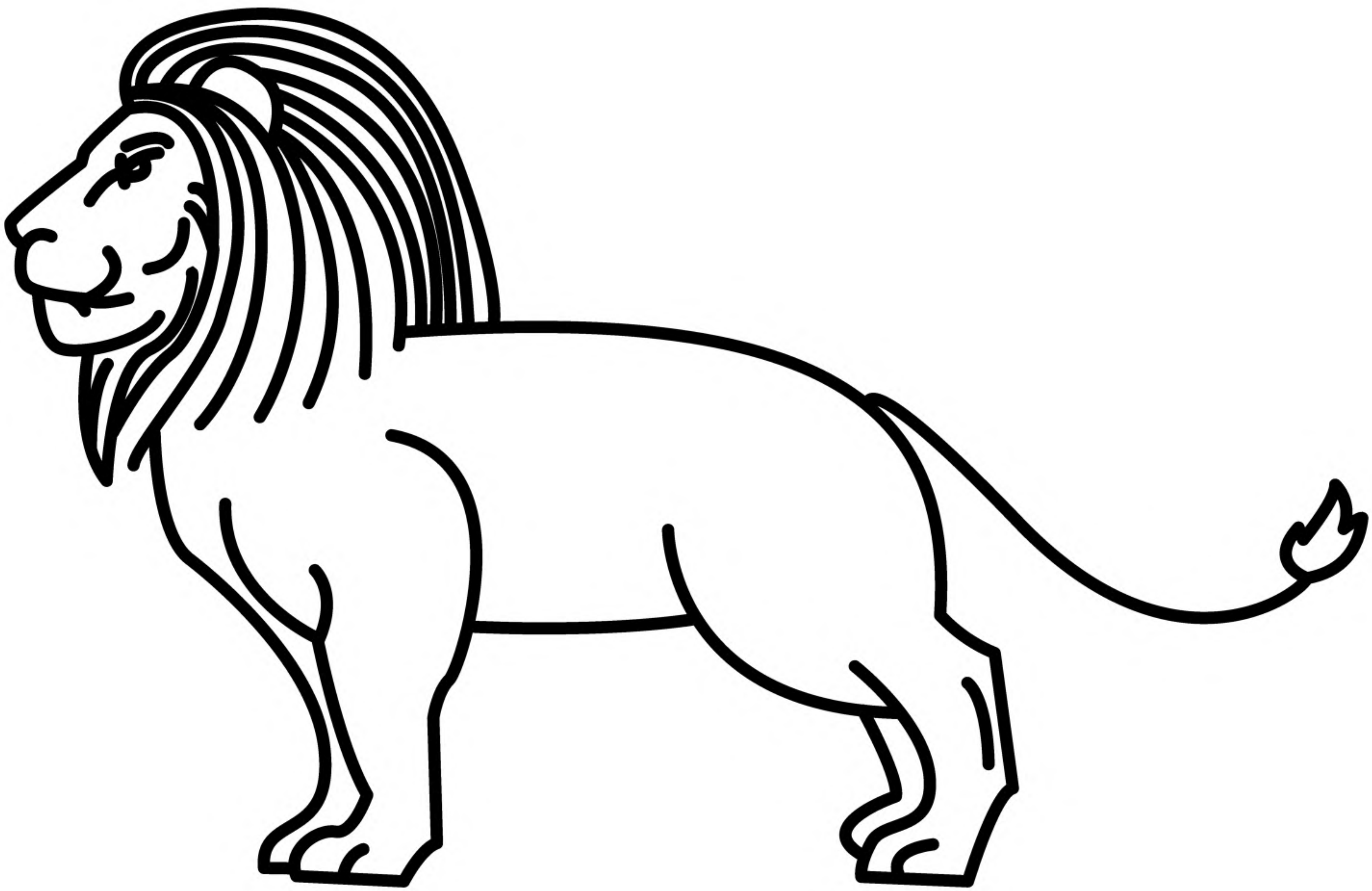

lion

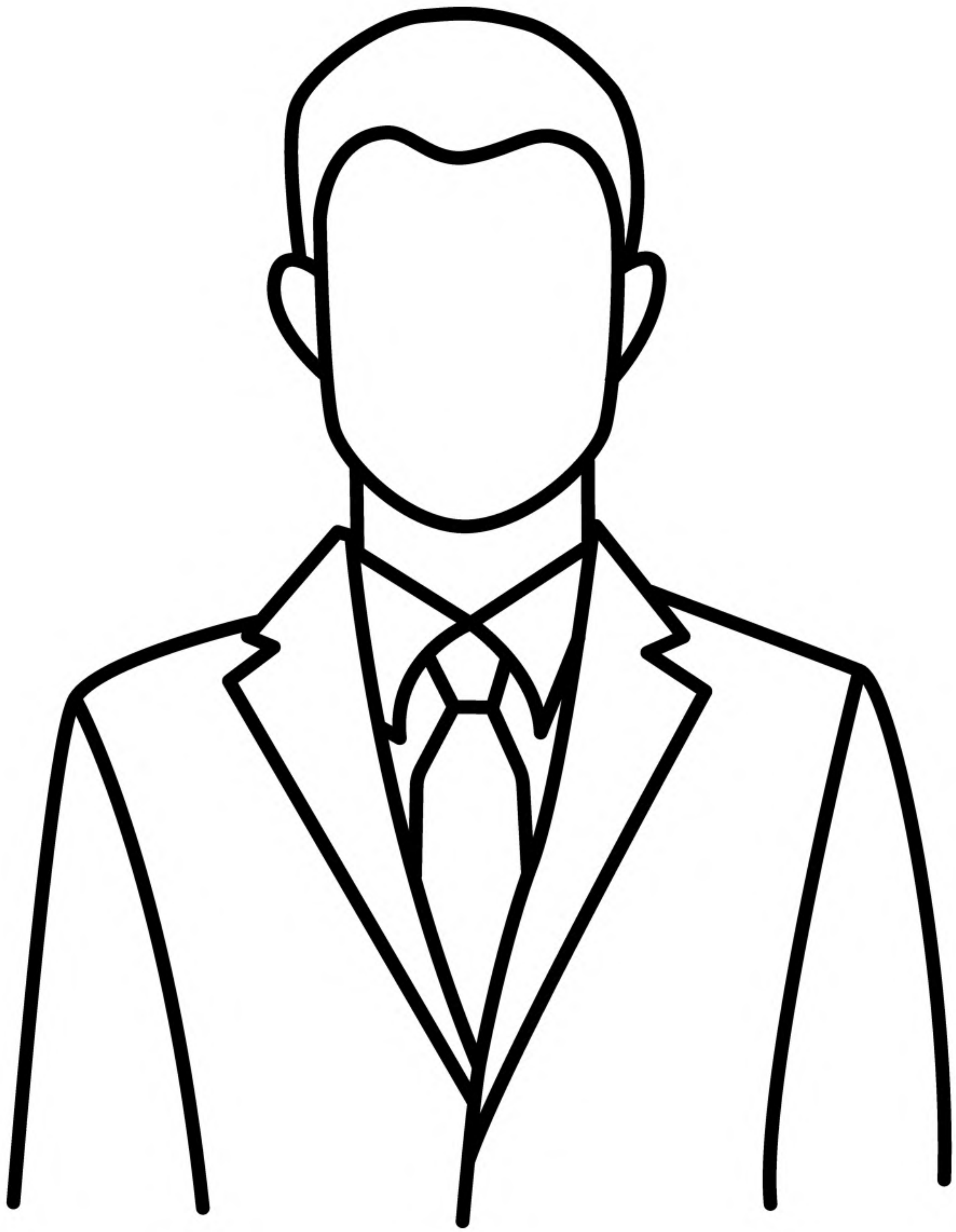

man

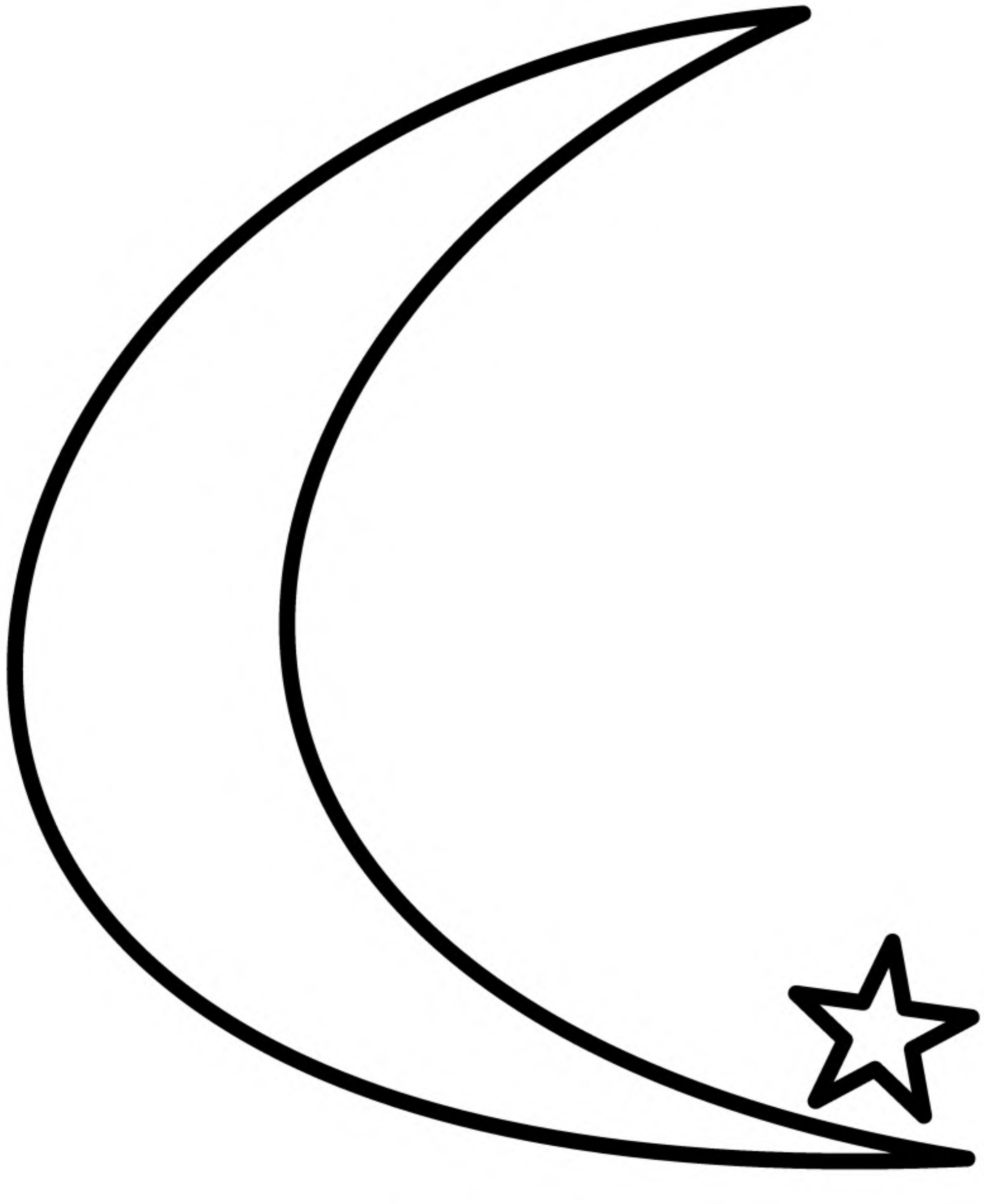

moon

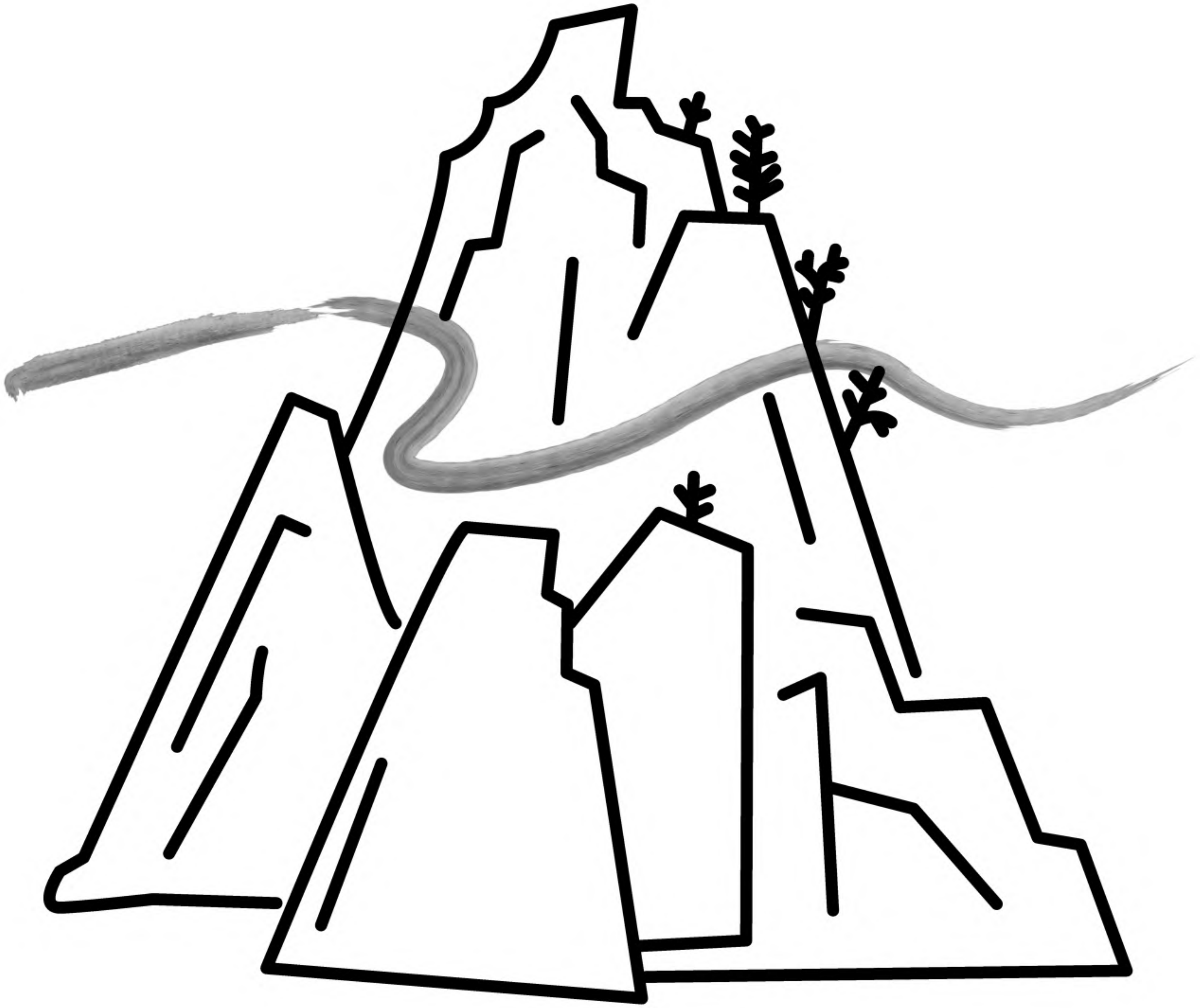

mountain

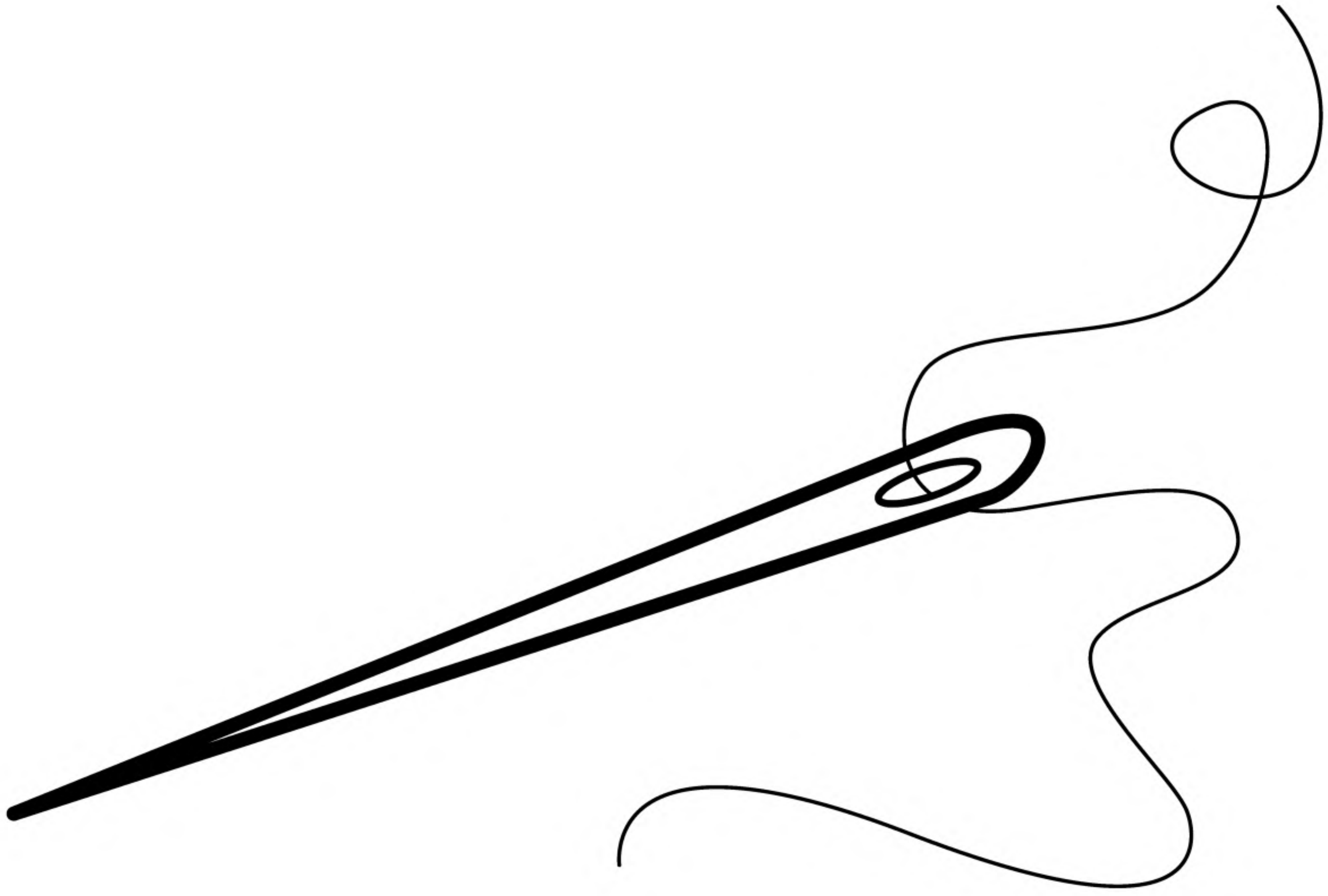

needle

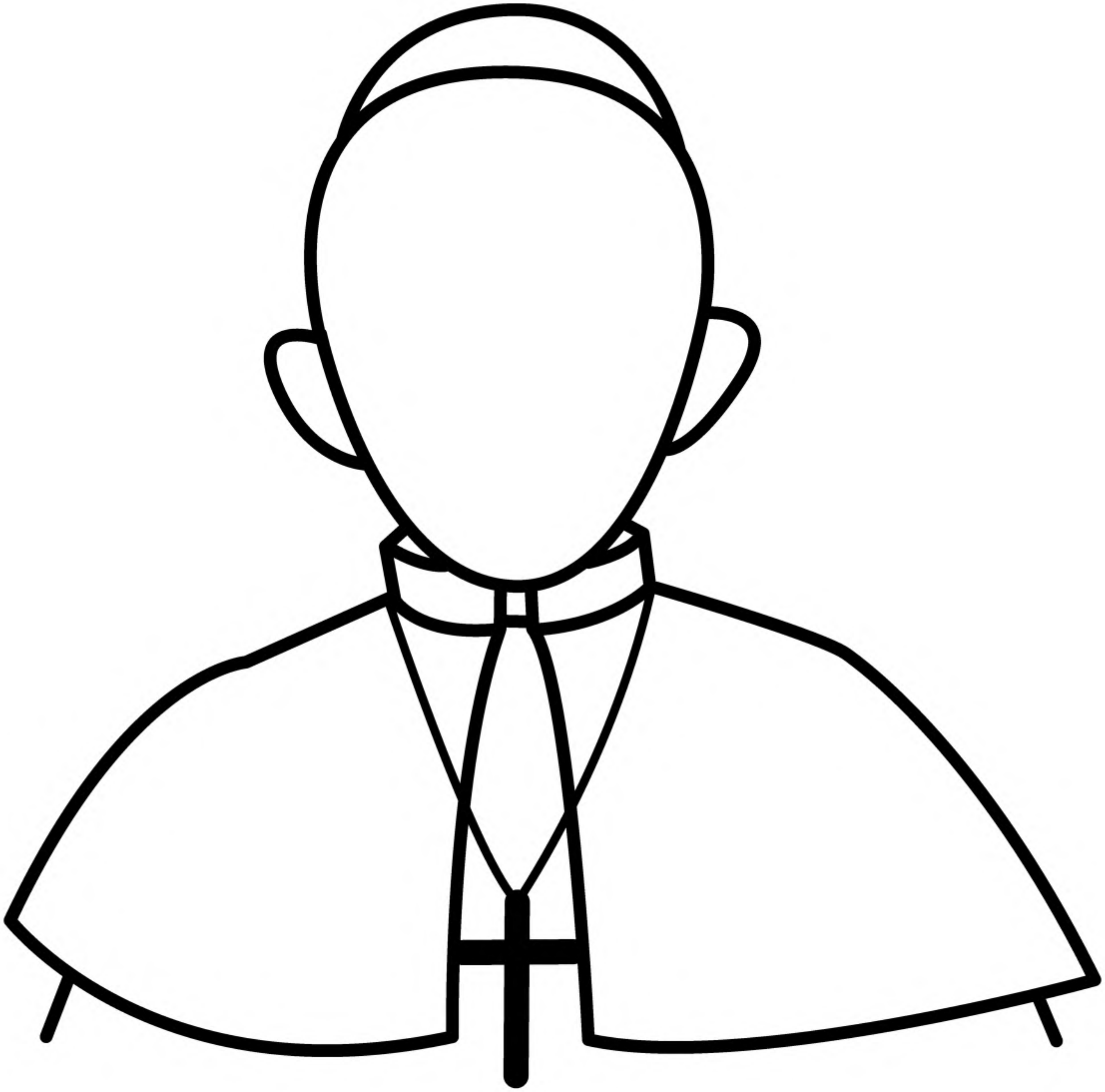

priest

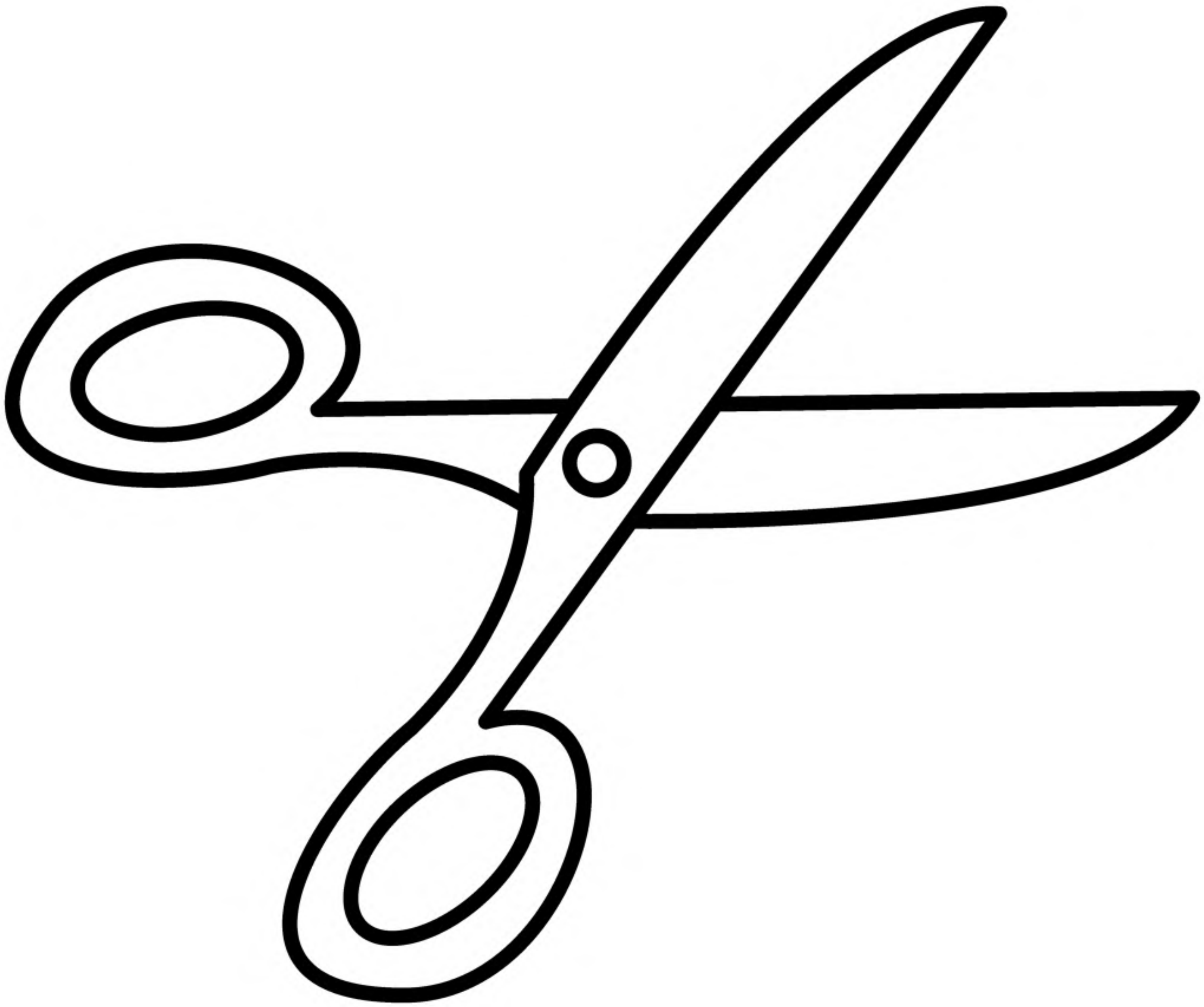

scissors

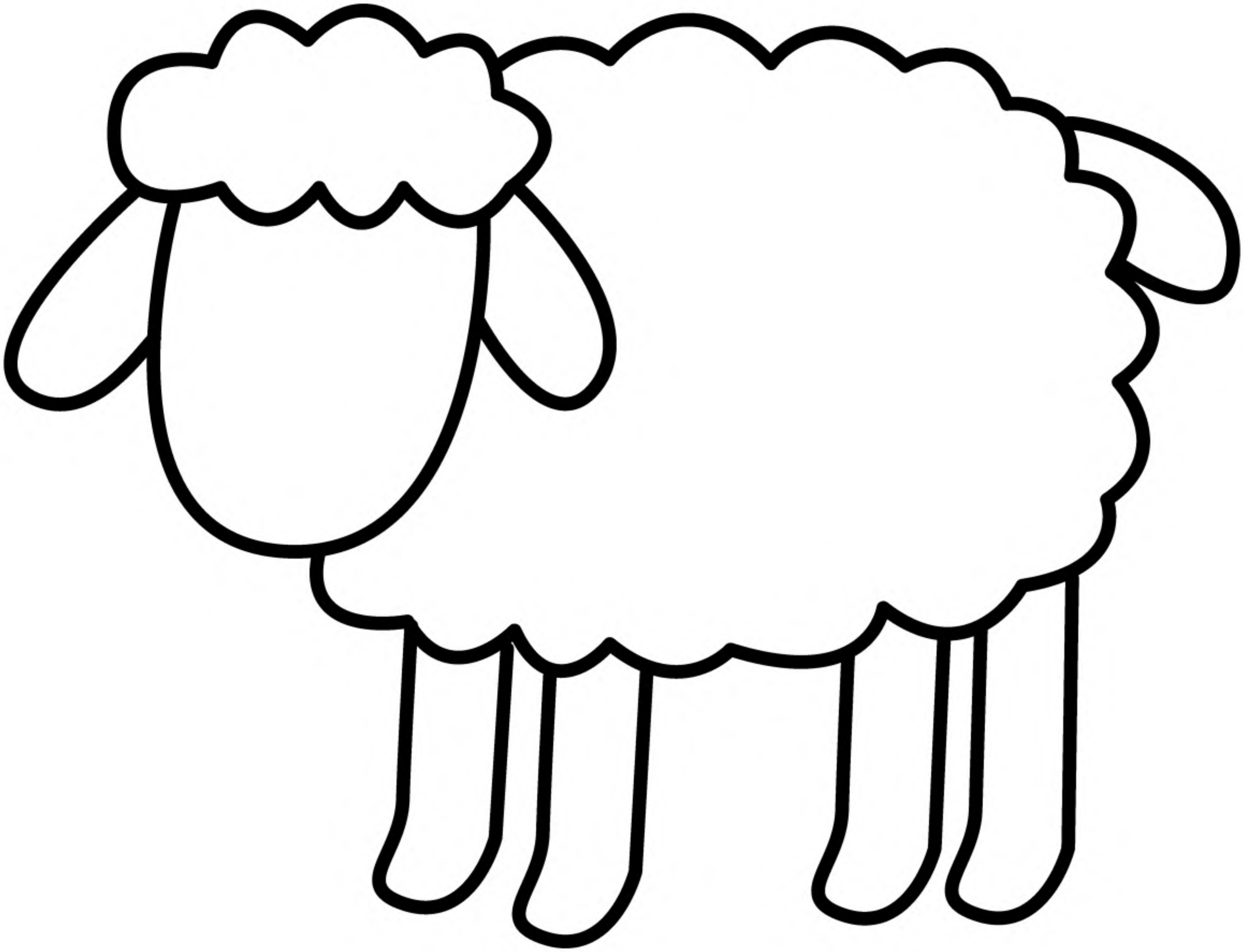

sheep

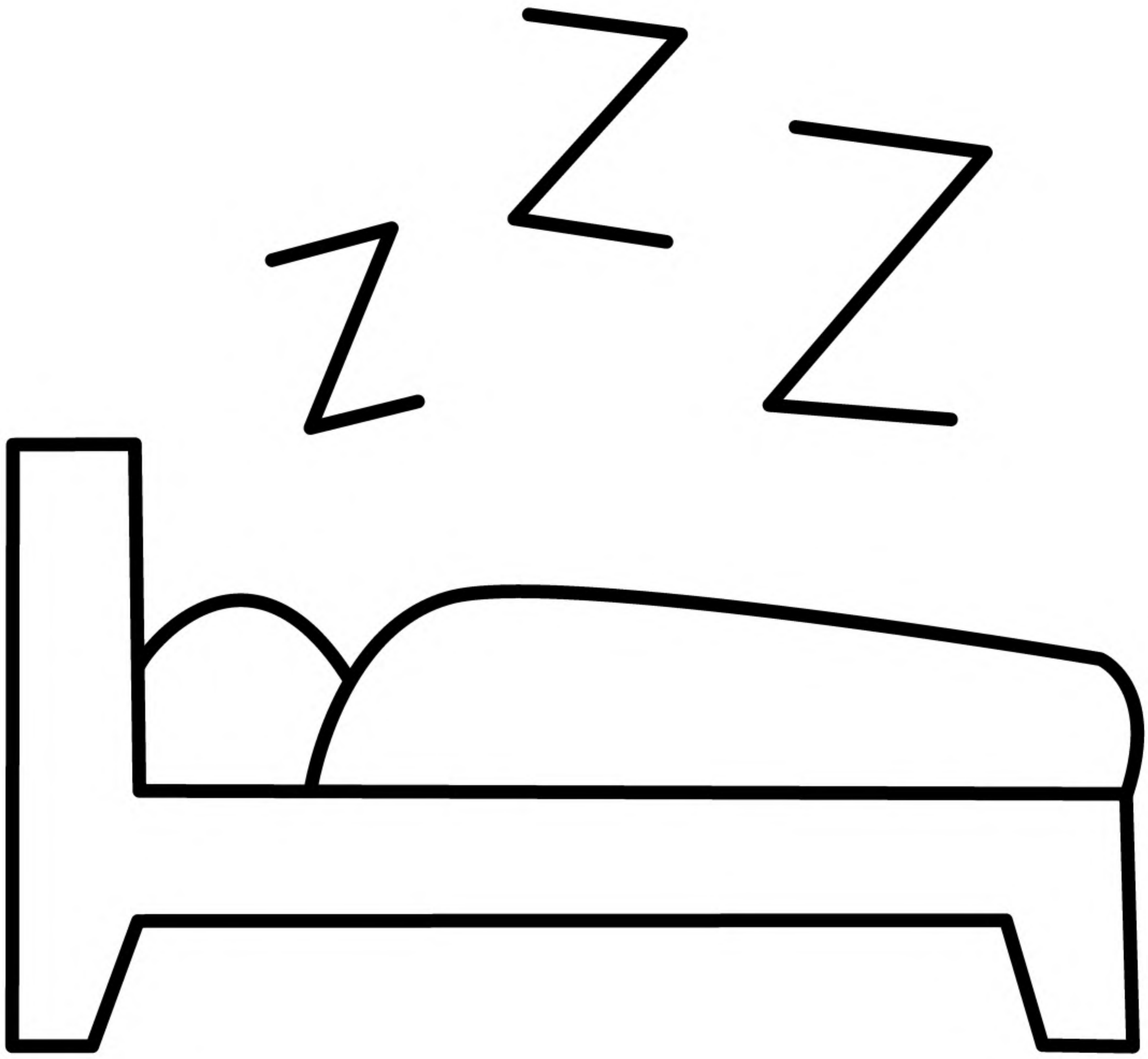

sleep

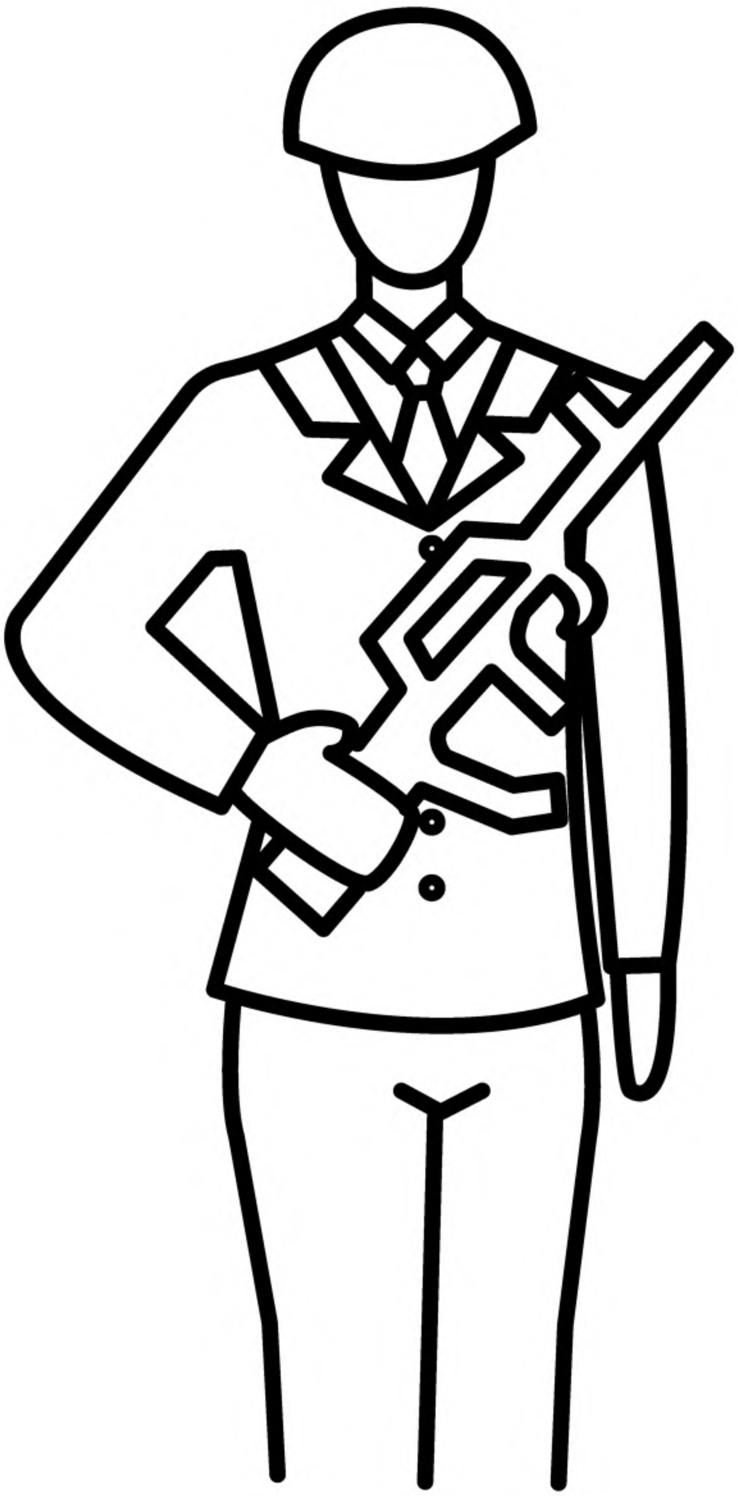

soldier

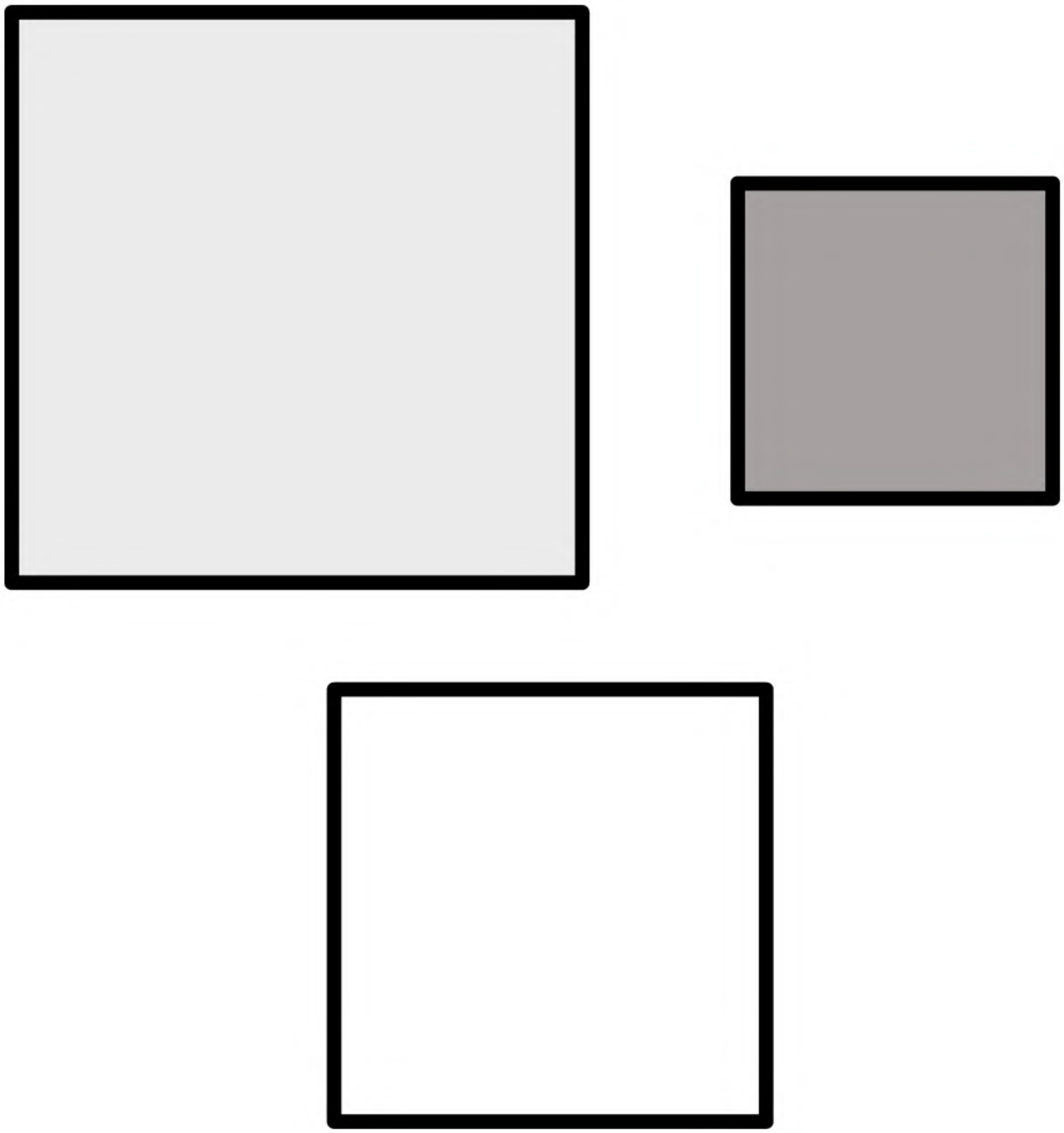

suqare

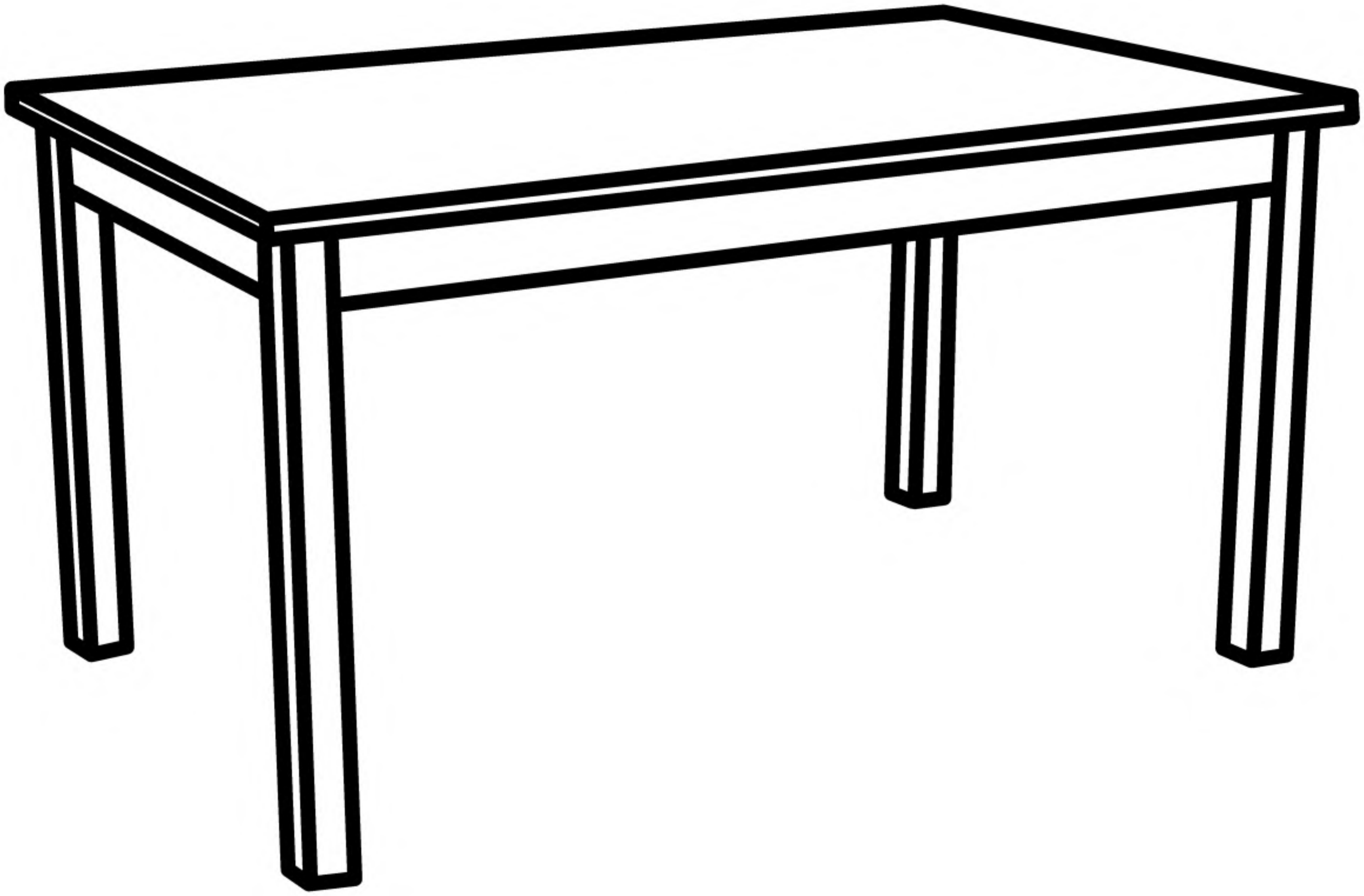

table

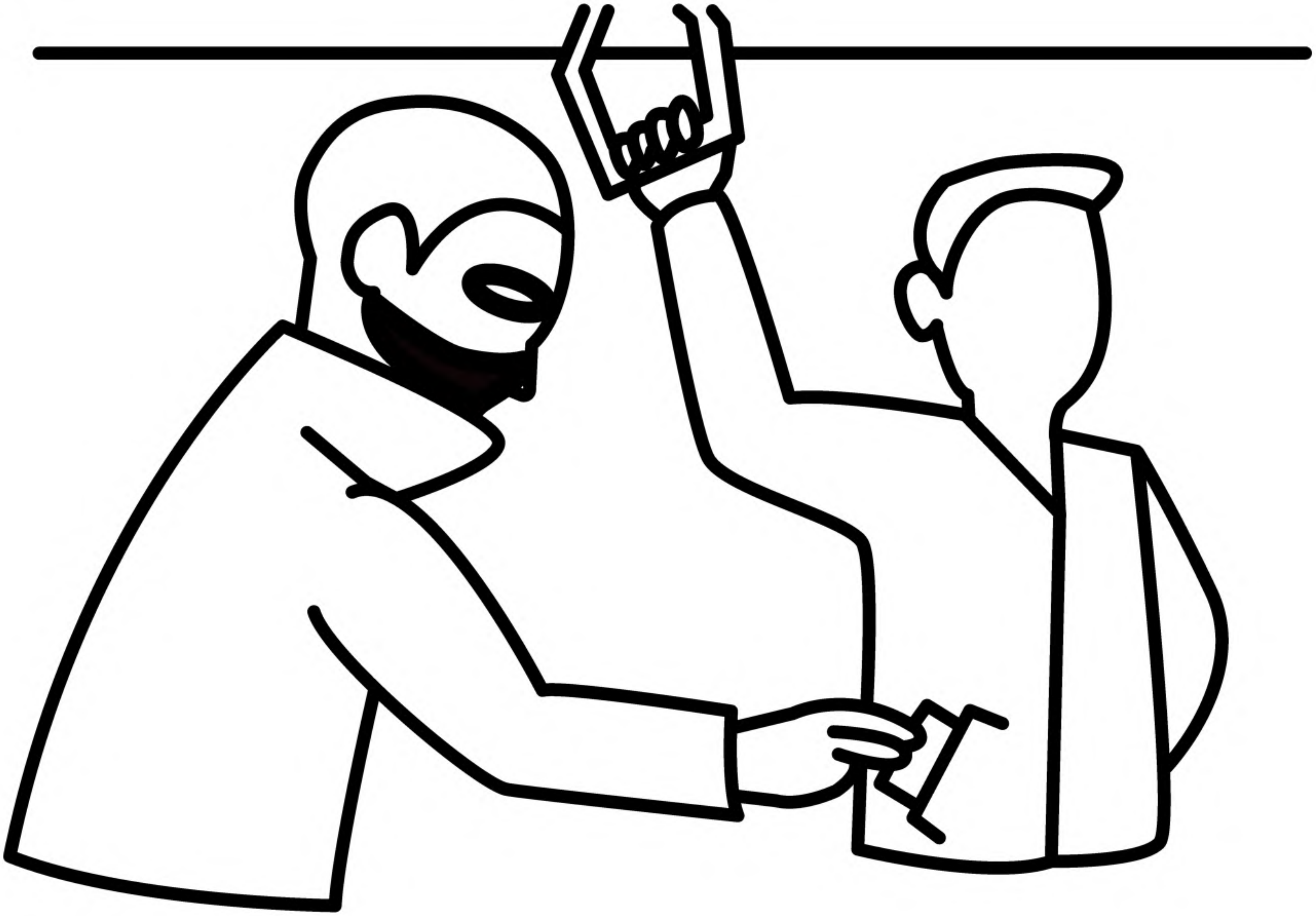

thief

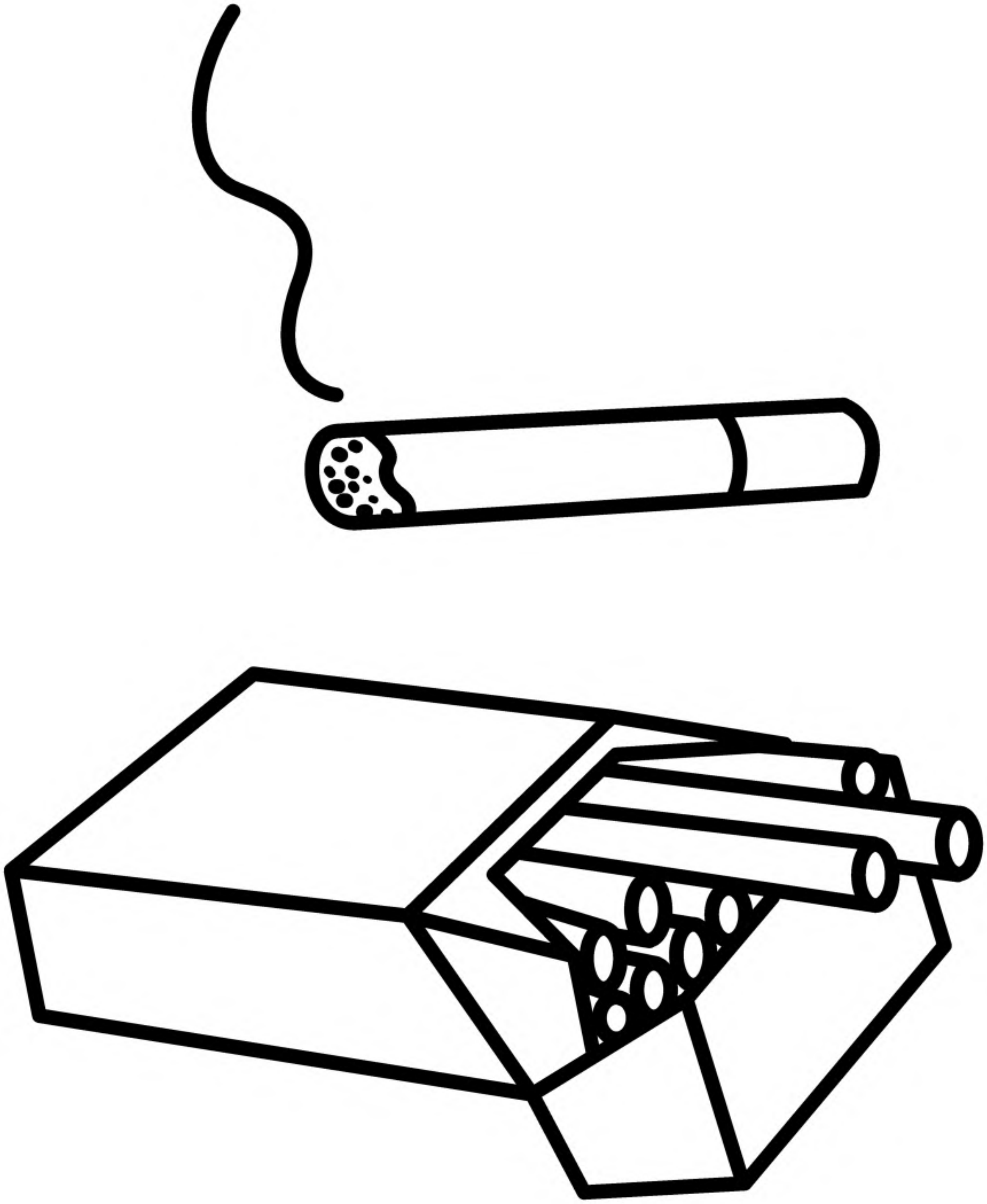

tobacco

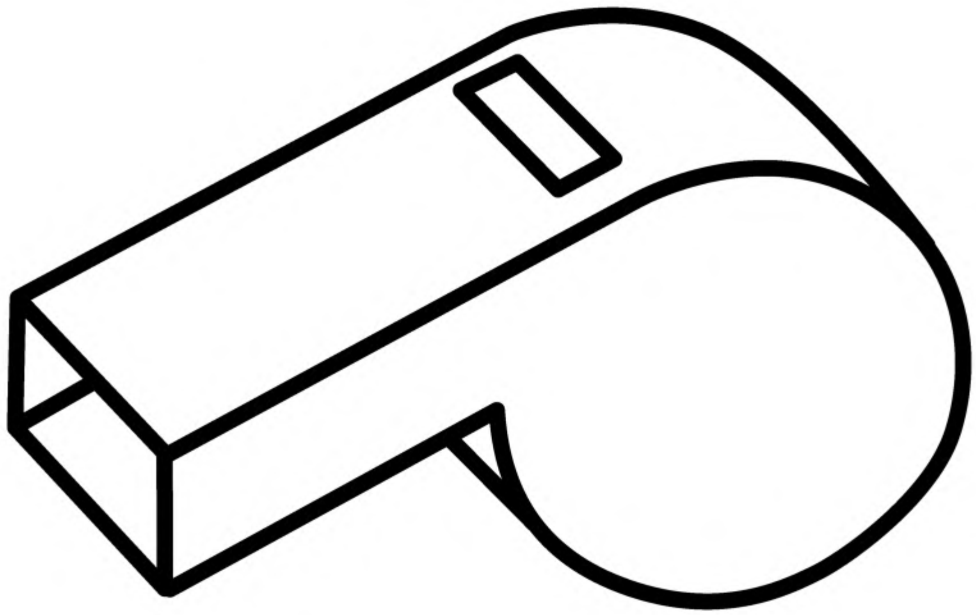

whistle

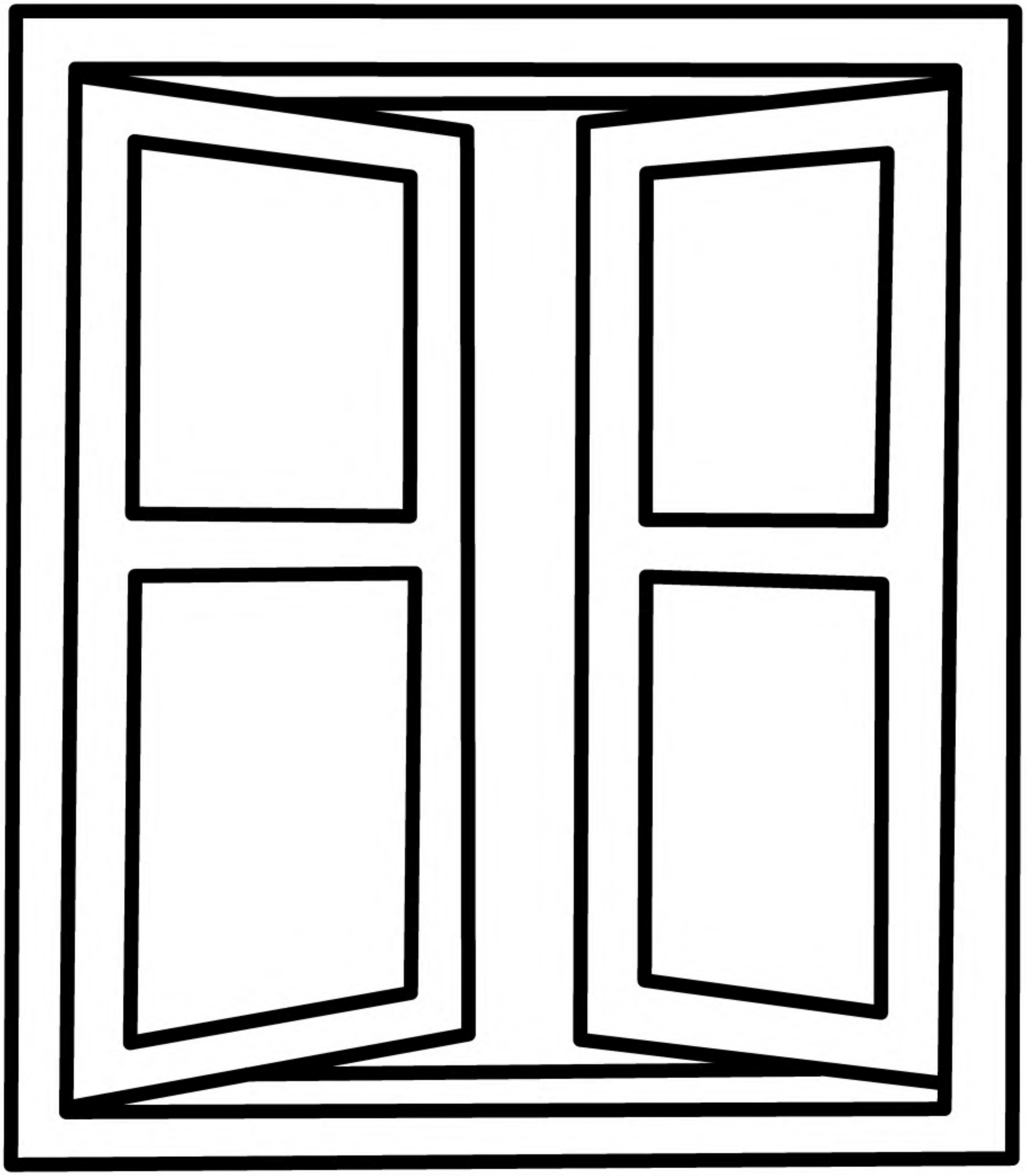

window

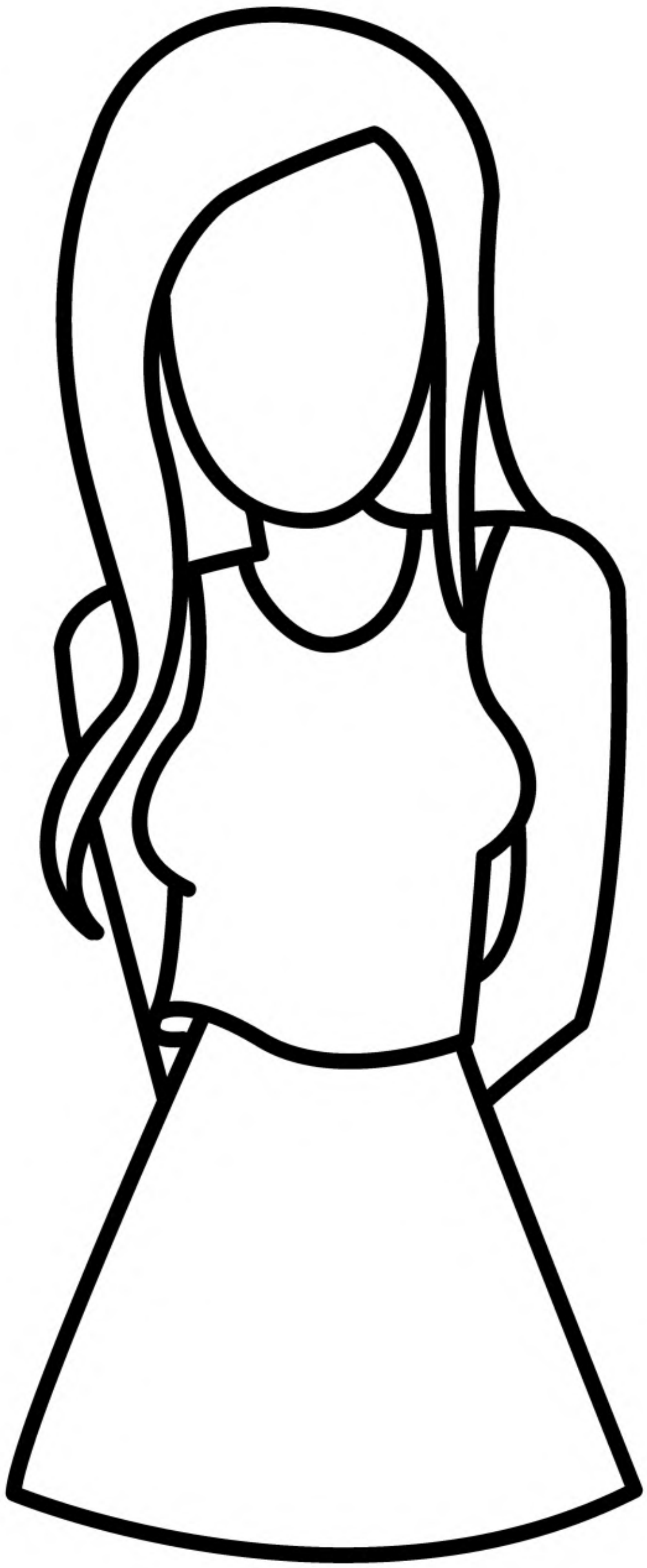

woman

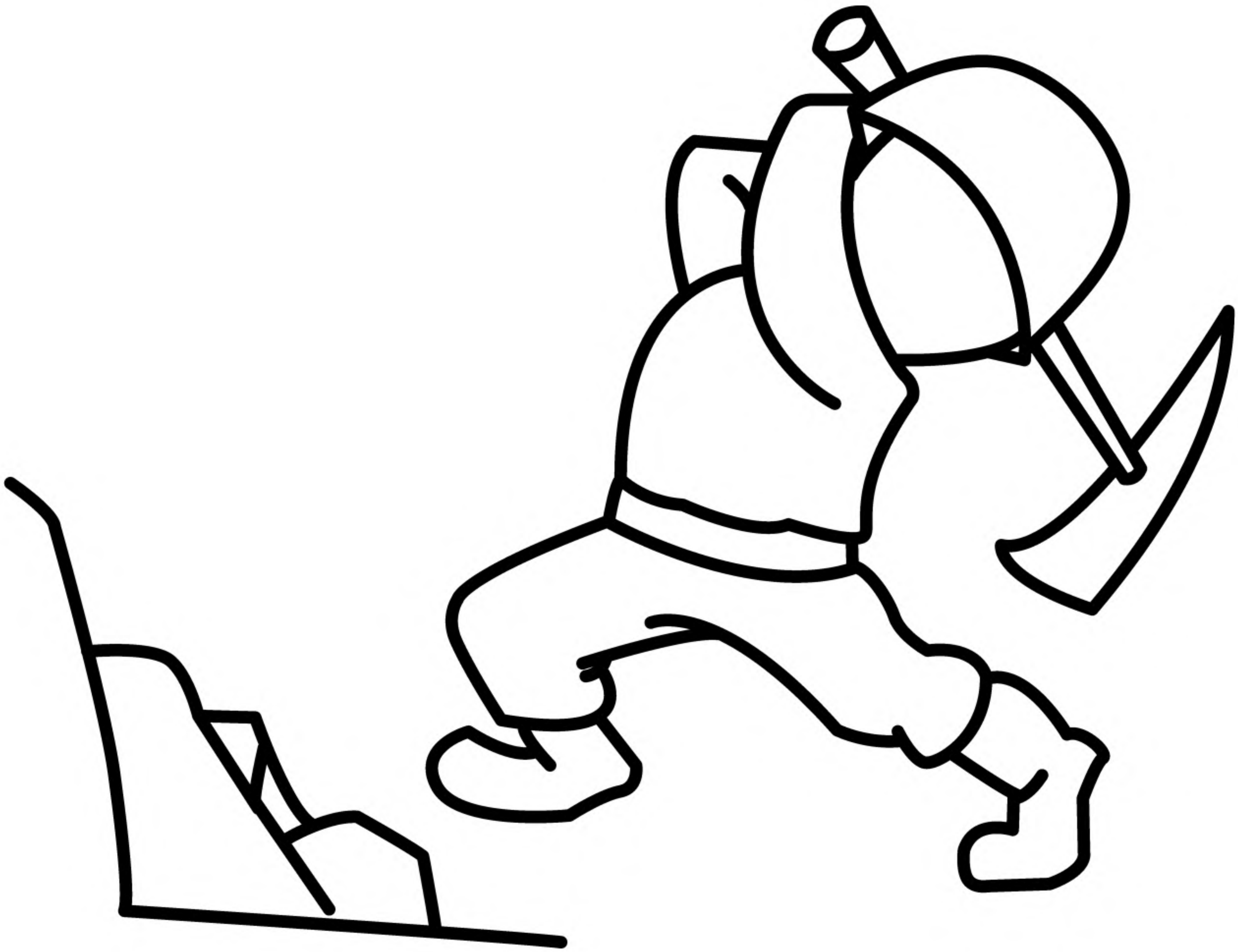

working
