# Supplementary material for: Brain hothubs and dark functional networks: correlation analysis between amplitude and connectivity for Broca’s aphasia
Source: PeerJ. 2020 Oct 1;8:e10057. doi: 10.7717/peerj.10057 (PMC7533062; doi:10.7717/peerj.10057)
Supplement: Table S1 [file peerj-08-10057-s002.docx]

**Table S1:**

**ROIs and corresponding functional modules in each hemisphere.**

| Functional Modules | ROIs | Abbreviations |
| --- | --- | --- |
| Attention | Lateralorbitofrontalgyrus_anterior | LatObtFrtGyr_Ant |
| Attention | Lateralorbitofrontalgyrus_posterior | LatObtFrtGyr_Pst |
| Attention | Superiorparietalgyrus_anterior | SupPariGyr_Ant |
| Attention | Superiorparietalgyrus_posterior | SupPariGyr_Pst |
| Auditory | Superiortemporalgyrus_anterior | SupTepGyr_Ant |
| Auditory | Superiortemporalgyrus_middle | SupTepGyr_Mid |
| Auditory | Superiortemporalgyrus_posterior | SupTepGyr_Pst |
| Auditory | Transversetemporalgyrus | TsvTepGyr |
| Cingulo-Opercular | Cingulategyrus_anterior | CingGyr_Ant |
| Cingulo-Opercular | Middlefrontalgyrus_anterior | MidFrtGyr_Ant |
| Cingulo-Opercular | Parsopercularis_inferior | ParsOpcu_Inf |
| Cingulo-Opercular | Parsopercularis_superior | ParsOpcu_Sup |
| Cingulo-Opercular | Parsorbitalis | ParsOrbitalis |
| Cingulo-Opercular | Supramarginalgyrus_anterior | SprmarGyr_Ant |
| Cingulo-Opercular | Supramarginalgyrus_posterior | SprmarGyr_Pst |
| Fronto-Parietal | Angulargyrus_anterior | AnguGyr_Ant |
| Fronto-Parietal | Angulargyrus_middle | AnguGyr_Mid |
| Fronto-Parietal | Angulargyrus_posterior | AnguGyr_Pst |
| Fronto-Parietal | Anteriororbito_frontalgyrus | AntObtFrtGyr |
| Fronto-Parietal | Gyrusrectus | GyrRectus |
| Fronto-Parietal | Insula_anterior | Insula_Ant |
| Fronto-Parietal | Insula_posterior | Insula_Pst |
| Fronto-Parietal | Middlefrontalgyrus_posterior | MidFrtGyr_Pst |
| Fronto-Parietal | Middleorbito-frontalgyrus | MidObtFrtGyr |
| Fronto-Parietal | Parstriangularis_anterior | ParsTriagu_Ant |
| Fronto-Parietal | Parstriangularis_middle | ParsTriagu_Mid |
| Fronto-Parietal | Parstriangularis_posterior | ParsTriagu_Pst |
| Fronto-Parietal | Posteriororbito-frontalgyrus | PstObtFrtGyr |
| Fronto-Parietal | Transversefrontalgyrus_lateral | TsvFrtGyr_Lat |
| Fronto-Parietal | Transversefrontalgyrus_mesial | TsvFrtGyr_Msl |
| Medial Default Mode | Cingulategyrus_middle | CingGyr_Mid |
| Medial Default Mode | Cingulategyrus_posterior | CingGyr_Pst |
| Medial Default Mode | Precuneus_inferior | PreCune_Inf |
| Medial Default Mode | Precuneus_superior | PreCune_Sup |
| Medial Default Mode | Subcallosalgyrus | SubcallosalGyr |
| Medial Default Mode | Superiorfrontalgyrus_anterior | SupFrtGyr_Ant |
| Medial Default Mode | Superiorfrontalgyrus_posterior | SupFrtGyr_Pst |
| Motor and Somatosensory | Paracentrallobule | ParaCentLob |
| Motor and Somatosensory | Postcentralgyrus_inferior | PostCentGyr_Inf |
| Motor and Somatosensory | Postcentralgyrus_superior | PostCentGyr_Sup |
| Motor and Somatosensory | Precentralgyrus_inferior | PreCentGyr_Inf |
| Motor and Somatosensory | Precentralgyrus_superior | PreCentGyr_Sup |
| Ventral Temporal Association | Fusiformgyrus_anterior | FusiGyr_Ant |
| Ventral Temporal Association | Fusiformgyrus_posterior | FusiGyr_Pst |
| Ventral Temporal Association | Inferiortemporalgyrus_anterior | InfTepGyr_Ant |
| Ventral Temporal Association | Inferiortemporalgyrus_middle | InfTepGyr_Mid |
| Ventral Temporal Association | Inferiortemporalgyrus_posterior | InfTepGyr_Pst |
| Ventral Temporal Association | Middletemporalgyrus_anterior | MidTepGyr_Ant |
| Ventral Temporal Association | Middletemporalgyrus_dorsoposterior | MidTepGyr_DsoPst |
| Ventral Temporal Association | Middletemporalgyrus_middle | MidTepGyr_Mid |
| Ventral Temporal Association | Middletemporalgyrus_ventroposterior | MidTepGyr_VenPst |
| Ventral Temporal Association | Parahippocampalgyrus | ParaHippoGyr |
| Ventral Temporal Association | Temporalpole | TepPole |
| Visual | Cuneus_anterior | Cune_Ant |
| Visual | Cuneus_posterior | Cune_Pst |
| Visual | Inferioroccipitalgyrus_anterior | InfOcciGyr_Ant |
| Visual | Inferioroccipitalgyrus_dorsoposterior | InfOcciGyr_DsoPst |
| Visual | Inferioroccipitalgyrus_ventroposterior | InfOcciGyr_VenPst |
| Visual | Lingualgyrus_anterior | LingualGyr_Ant |
| Visual | Lingualgyrus_posterior | LingualGyr_Pst |
| Visual | Middleoccipitalgyrus_dorsoanterior | MidOcciGyr_DsoAnt |
| Visual | Middleoccipitalgyrus_posterior | MidOcciGyr_Pst |
| Visual | Middleoccipitalgyrus_ventroanterior | MidOcciGyr_VenAnt |
| Visual | Superioroccipitalgyrus_inferior | SupOcciGyr_Inf |
| Visual | Superioroccipitalgyrus_superior | SupOcciGyr_Sup |
