# Supplementary material for: Brain hothubs and dark functional networks: correlation analysis between amplitude and connectivity for Broca’s aphasia
Source: PeerJ. 2020 Oct 1;8:e10057. doi: 10.7717/peerj.10057 (PMC7533062; doi:10.7717/peerj.10057)
Supplement: Table S2 — This table showed the hothubs in the two groups. The citations in this table reported that the regions are activated in picture-naming. [file peerj-08-10057-s003.docx]

**Table S2:**

**Hothubs in the two groups and reports supporting them as identifying specific regions.**

| **ROIs** | **References** |
| --- | --- |
| AnguGyr_Ant_L | (Bowyer et al., 2004; Blanco-Elorrieta et al., 2018) |
| AnguGyr_Mid_L | (Bowyer et al., 2004; Blanco-Elorrieta et al., 2018) |
| AnguGyr_Mid_R | (Nardo et al., 2017) |
| AnguGyr_Pst_L | (Bowyer et al., 2004; Blanco-Elorrieta et al., 2018) |
| AnguGyr_Pst_R | (Nardo et al., 2017) |
| AntObtFrtGyr_R | (Piai et al., 2015) |
| CingGyr_Ant_R | (Moore & Price, 1999) |
| CingGyr_Mid_L | (Garn, Allen & Larsen, 2009) |
| Cune_Ant_L | (Whatmough et al., 2002; Chouinard & Goodale, 2010; Laaksonen et al., 2012; Hyder et al., 2015; Miozzo, Pulvermüller & Hauk, 2015; Liljeström et al., 2015) |
| Cune_Ant_R | (Whatmough et al., 2002; Chouinard & Goodale, 2010) |
| Cune_Pst_L | (Whatmough et al., 2002; Chouinard & Goodale, 2010; Laaksonen et al., 2012; Hyder et al., 2015; Miozzo, Pulvermüller & Hauk, 2015; Liljeström et al., 2015) |
| Cune_Pst_R | (Whatmough et al., 2002; Chouinard & Goodale, 2010) |
| FusiGyr_Pst_R | - |
| GyrRectus_L | (Race et al., 2013) |
| GyrRectus_R | (Chouinard & Goodale, 2010; Takahashi et al., 2017) |
| InfOcciGyr_Ant_L | (Vihla, Laine & Salmelin, 2006; Laaksonen et al., 2012; Hyder et al., 2015; Miozzo, Pulvermüller & Hauk, 2015; Liljeström et al., 2015) |
| InfOcciGyr_Ant_R | (Vihla, Laine & Salmelin, 2006; Laaksonen et al., 2012; Hyder et al., 2015; Miozzo, Pulvermüller & Hauk, 2015; Liljeström et al., 2015) |
| InfOcciGyr_DsoPst_L | (Vihla, Laine & Salmelin, 2006; Laaksonen et al., 2012; Hyder et al., 2015; Miozzo, Pulvermüller & Hauk, 2015; Liljeström et al., 2015) |
| InfOcciGyr_DsoPst_R | (Vihla, Laine & Salmelin, 2006; Laaksonen et al., 2012; Hyder et al., 2015; Miozzo, Pulvermüller & Hauk, 2015; Liljeström et al., 2015) |
| InfOcciGyr_VenPst_L | (Vihla, Laine & Salmelin, 2006; Laaksonen et al., 2012; Hyder et al., 2015; Miozzo, Pulvermüller & Hauk, 2015; Liljeström et al., 2015) |
| InfOcciGyr_VenPst_R | (Vihla, Laine & Salmelin, 2006; Laaksonen et al., 2012; Hyder et al., 2015; Miozzo, Pulvermüller & Hauk, 2015; Liljeström et al., 2015) |
| InfTepGyr_Ant_L | (Miozzo, Pulvermüller & Hauk, 2015; Blanco-Elorrieta et al., 2018; Zhang et al., 2018) |
| InfTepGyr_Mid_R | (Zhang et al., 2018) |
| Insula_Ant_L | (Yang et al., 2018) |
| Insula_Ant_R | (Yang et al., 2018) |
| LatObtFrtGyr_Ant_R | (Piai et al., 2015) |
| LatObtFrtGyr_Pst_L | (Piai et al., 2015) |
| LatObtFrtGyr_Pst_R | (Piai et al., 2015) |
| LingualGyr_Ant_R | (Whatmough et al., 2002) |
| LingualGyr_Pst_R | (Whatmough et al., 2002) |
| MidFrtGyr_Ant_R | (Yang et al., 2018) |
| MidFrtGyr_Pst_R | (Yang et al., 2018) |
| MidObtFrtGyr_L | (Piai et al., 2015) |
| MidObtFrtGyr_R | (Piai et al., 2015) |
| MidOcciGyr_DsoAnt_L | (Vihla, Laine & Salmelin, 2006; Laaksonen et al., 2012; Hyder et al., 2015; Miozzo, Pulvermüller & Hauk, 2015; Liljeström et al., 2015) |
| MidOcciGyr_Pst_R | (Vihla, Laine & Salmelin, 2006; Laaksonen et al., 2012; Hyder et al., 2015; Miozzo, Pulvermüller & Hauk, 2015; Liljeström et al., 2015) |
| MidOcciGyr_VenAnt_L | (Vihla, Laine & Salmelin, 2006; Laaksonen et al., 2012; Hyder et al., 2015; Miozzo, Pulvermüller & Hauk, 2015; Liljeström et al., 2015) |
| MidOcciGyr_VenAnt_R | (Vihla, Laine & Salmelin, 2006; Laaksonen et al., 2012; Hyder et al., 2015; Miozzo, Pulvermüller & Hauk, 2015; Liljeström et al., 2015) |
| MidTepGyr_Ant_L | (Levelt et al., 1998; Maess et al., 2002; Miozzo, Pulvermüller & Hauk, 2015; Blanco-Elorrieta et al., 2018; Zhang et al., 2018) |
| MidTepGyr_Ant_R | (Blanco-Elorrieta et al., 2018; Zhang et al., 2018) |
| MidTepGyr_DsoPst_L | (Levelt et al., 1998; Maess et al., 2002; Miozzo, Pulvermüller & Hauk, 2015; Zhang et al., 2018) |
| MidTepGyr_VenPst_L | (Levelt et al., 1998; Maess et al., 2002; Miozzo, Pulvermüller & Hauk, 2015; Zhang et al., 2018) |
| ParsOpcu_Inf_R | (Bowyer et al., 2005; Vihla, Laine & Salmelin, 2006; Hyder et al., 2015; Yang et al., 2018) |
| ParsOpcu_Sup_L | (Bowyer et al., 2004, 2005; Vihla, Laine & Salmelin, 2006; Hyder et al., 2015; Piai et al., 2015; Shinshi et al., 2015; Miozzo, Pulvermüller & Hauk, 2015; Liljeström et al., 2015; Blanco-Elorrieta et al., 2018) |
| ParsOrbitalis_R | (Bowyer et al., 2005; Vihla, Laine & Salmelin, 2006; Hyder et al., 2015; Yang et al., 2018) |
| ParsTriagu_Ant_L | (Bowyer et al., 2004, 2005; Vihla, Laine & Salmelin, 2006; Hyder et al., 2015; Piai et al., 2015; Shinshi et al., 2015; Miozzo, Pulvermüller & Hauk, 2015; Liljeström et al., 2015; Blanco-Elorrieta et al., 2018) |
| ParsTriagu_Ant_R | (Bowyer et al., 2005; Vihla, Laine & Salmelin, 2006; Hyder et al., 2015; Yang et al., 2018) |
| ParsTriagu_Mid_L | (Bowyer et al., 2004, 2005; Vihla, Laine & Salmelin, 2006; Hyder et al., 2015; Piai et al., 2015; Shinshi et al., 2015; Miozzo, Pulvermüller & Hauk, 2015; Liljeström et al., 2015; Blanco-Elorrieta et al., 2018) |
| ParsTriagu_Pst_L | (Bowyer et al., 2004, 2005; Vihla, Laine & Salmelin, 2006; Hyder et al., 2015; Piai et al., 2015; Shinshi et al., 2015; Miozzo, Pulvermüller & Hauk, 2015; Liljeström et al., 2015; Blanco-Elorrieta et al., 2018) |
| PostCentGyr_Inf_L | (Hansen, McMahon & de Zubicaray, 2019) |
| PostCentGyr_Sup_L | (Hansen, McMahon & de Zubicaray, 2019) |
| PreCentGyr_Inf_L | (Zhang et al., 2018) |
| PreCentGyr_Inf_R | (Zhang et al., 2018) |
| PreCentGyr_Sup_L | (Zhang et al., 2018) |
| PreCune_Inf_L | (Vitali et al., 2005; Basso et al., 2013; Wang et al., 2019) |
| PreCune_Inf_R | (Heath et al., 2012) |
| PreCune_Sup_L | (Vihla, Laine & Salmelin, 2006; Laaksonen et al., 2012; Hyder et al., 2015; Miozzo, Pulvermüller & Hauk, 2015; Liljeström et al., 2015) |
| PreCune_Sup_R | (Heath et al., 2012; Laaksonen et al., 2012; Hyder et al., 2015; Miozzo, Pulvermüller & Hauk, 2015; Liljeström et al., 2015) |
| PstObtFrtGyr_L | (Piai et al., 2015) |
| PstObtFrtGyr_R | (Piai et al., 2015) |
| SprmarGyr_Pst_R | (Bowyer et al., 2005; Yang et al., 2018) |
| SubcallosalGyr_R | (Takahashi et al., 2017) |
| SupFrtGyr_Ant_L | (Piai et al., 2015) |
| SupFrtGyr_Pst_R | (Piai et al., 2015) |
| SupOcciGyr_Inf_L | (Laaksonen et al., 2012; Hyder et al., 2015; Miozzo, Pulvermüller & Hauk, 2015; Liljeström et al., 2015) |
| SupOcciGyr_Inf_R | (Laaksonen et al., 2012; Hyder et al., 2015; Miozzo, Pulvermüller & Hauk, 2015; Liljeström et al., 2015) |
| SupOcciGyr_Sup_L | (Laaksonen et al., 2012; Hyder et al., 2015; Miozzo, Pulvermüller & Hauk, 2015; Liljeström et al., 2015) |
| SupOcciGyr_Sup_R | (Laaksonen et al., 2012; Hyder et al., 2015; Miozzo, Pulvermüller & Hauk, 2015; Liljeström et al., 2015) |
| SupPariGyr_Ant_R | (Levelt et al., 1998) |
| SupPariGyr_Pst_L | (Wu et al., 2011; Hansen, McMahon & de Zubicaray, 2019) |
| SupPariGyr_Pst_R | (Levelt et al., 1998) |
| SupTepGyr_Ant_L | (Levelt et al., 1998; Maess et al., 2002; Bowyer et al., 2004, 2005; Blanco-Elorrieta et al., 2018; Zhang et al., 2018) |
| SupTepGyr_Pst_L | (Levelt et al., 1998; Maess et al., 2002; Bowyer et al., 2004, 2005; Vihla, Laine & Salmelin, 2006; Brooks & Cid de Garcia, 2015; Miozzo, Pulvermüller & Hauk, 2015; Liljeström et al., 2015; Zhang et al., 2018) |
| TepPole_L | (Levelt et al., 1998; Maess et al., 2002; Zhang et al., 2018) |
| TsvFrtGyr_Lat_R | (Piai et al., 2015) |
| TsvFrtGyr_Msl_R | (Piai et al., 2015) |
| TsvTepGyr_L | (Levelt et al., 1998; Zhang et al., 2018) |

**REFERENCES**

Basso G, Magon S, Reggiani F, Capasso R, Monittola G, Yang F-J, Miceli G. 2013. Distinguishable neurofunctional effects of task practice and item practice in picture naming: A BOLD fMRI study in healthy subjects. *Brain and Language* 126:302–313. DOI: 10.1016/j.bandl.2013.07.002.

Blanco-Elorrieta E, Ferreira VS, Del Prato P, Pylkkänen L. 2018. The priming of basic combinatory responses in MEG. *Cognition* 170:49–63. DOI: 10.1016/j.cognition.2017.09.010.

Bowyer SM, Moran JE, Mason KM, Constantinou JE, Smith BJ, Barkley GL, Tepley N. 2004. MEG localization of language-specific cortex utilizing MR-FOCUSS. *Neurology* 62:2247–2255. DOI: 10.1212/01.WNL.0000130385.21160.7A.

Bowyer SM, Moran JE, Weiland BJ, Mason KM, Greenwald ML, Smith BJ, Barkley GL, Tepley N. 2005. Language laterality determined by MEG mapping with MR-FOCUSS. *Epilepsy & Behavior* 6:235–241. DOI: 10.1016/j.yebeh.2004.12.002.

Brooks TL, Cid de Garcia D. 2015. Evidence for morphological composition in compound words using MEG. *Frontiers in Human Neuroscience* 9:215. DOI: 10.3389/fnhum.2015.00215.

Chouinard PA, Goodale MA. 2010. Category-specific neural processing for naming pictures of animals and naming pictures of tools: An ALE meta-analysis. *Neuropsychologia* 48:409–418. DOI: 10.1016/j.neuropsychologia.2009.09.032.

Garn CL, Allen MD, Larsen JD. 2009. An fMRI study of sex differences in brain activation during object naming. *Cortex* 45:610–618. DOI: 10.1016/j.cortex.2008.02.004.

Hansen SJ, McMahon KL, de Zubicaray GI. 2019. Neural mechanisms for monitoring and halting of spoken word production. *Journal of Cognitive Neuroscience* 31:1946–1957. DOI: 10.1162/jocn_a_01462.

Heath S, McMahon K, Nickels L, Angwin A, MacDonald A, van Hees S, Johnson K, Copland D. 2012. Priming picture naming with a semantic task: an fMRI investigation. *PLoS One* 7:e32809. DOI: 10.1371/journal.pone.0032809.

Hyder R, Kamel N, Boon TT, Reza F. 2015. Mapping of language brain areas in patients with brain tumors. In: *Proceedings of the 37th Annual International Conference of the IEEE Engineering in Medicine and Biology Society (EMBC)*. Milan, Italy: IEEE, 626–629. DOI: 10.1109/EMBC.2015.7318440.

Laaksonen H, Kujala J, Hultén A, Liljeström M, Salmelin R. 2012. MEG evoked responses and rhythmic activity provide spatiotemporally complementary measures of neural activity in language production. *NeuroImage* 60:29–36. DOI: 10.1016/j.neuroimage.2011.11.087.

Levelt WJ, Praamstra P, Meyer AS, Helenius P, Salmelin R. 1998. An MEG study of picture naming. *Journal of Cognitive Neuroscience* 10:553–567. DOI: 10.1162/089892998562960.

Liljeström M, Stevenson C, Kujala J, Salmelin R. 2015. Task- and stimulus-related cortical networks in language production: Exploring similarity of MEG- and fMRI-derived functional connectivity. *NeuroImage* 120:75–87. DOI: 10.1016/j.neuroimage.2015.07.017.

Maess B, Friederici AD, Damian M, Meyer AS, Levelt WJM. 2002. Semantic category interference in overt picture naming: Sharpening current density localization by PCA. *Journal of Cognitive Neuroscience* 14:455–462. DOI: 10.1162/089892902317361967.

Miozzo M, Pulvermüller F, Hauk O. 2015. Early parallel activation of semantics and phonology in picture naming: Evidence from a multiple linear regression MEG study. *Cerebral Cortex* 25:3343–3355. DOI: 10.1093/cercor/bhu137.

Moore CJ, Price CJ. 1999. Three distinct ventral occipitotemporal regions for reading and object naming. *NeuroImage* 10:181–192. DOI: 10.1006/nimg.1999.0450.

Nardo D, Holland R, Leff AP, Price CJ, Crinion JT. 2017. Less is more: Neural mechanisms underlying anomia treatment in chronic aphasic patients. *Brain* 140:3039–3054. DOI: 10.1093/brain/awx234.

Piai V, Roelofs A, Rommers J, Dahlslätt K, Maris E. 2015. Withholding planned speech is reflected in synchronized beta-band oscillations. *Frontiers in Human Neuroscience* 9:549. DOI: 10.3389/fnhum.2015.00549.

Race DS, Tsapkini K, Crinion J, Newhart M, Davis C, Gomez Y, Hillis AE, Faria AV. 2013. An area essential for linking word meanings to word forms: Evidence from primary progressive aphasia. *Brain and Language* 127:167–176. DOI: 10.1016/j.bandl.2013.09.004.

Shinshi M, Yanagisawa T, Hirata M, Goto T, Sugata H, Araki T, Okamura Y, Hasegawa Y, Ihara AS, Yorifuji S. 2015. Temporospatial identification of language-related cortical function by a combination of transcranial magnetic stimulation and magnetoencephalography. *Brain and Behavior* 5:e00317. DOI: 10.1002/brb3.317.

Takahashi M, Oda Y, Okubo T, Shirayama Y. 2017. Relationships between cognitive impairment on ADAS-cog and regional cerebral blood flow using SPECT in late-onset Alzheimer’s disease. *Journal of Neural Transmission* 124:1109–1121. DOI: 10.1007/s00702-017-1734-7.

Vihla M, Laine M, Salmelin R. 2006. Cortical dynamics of visual/semantic vs. phonological analysis in picture confrontation. *Neuroimage* 33:732–738. DOI: 10.1016/j.neuroimage.2006.06.040.

Vitali P, Abutalebi J, Tettamanti M, Rowe J, Scifo P, Fazio F, Cappa SF, Perani D. 2005. Generating animal and tool names: An fMRI study of effective connectivity. *Brain and Language* 93:32–45. DOI: 10.1016/j.bandl.2004.08.005.

Wang Z, Liu F, Sun Y, Li J, Wang F, Lu Z. 2019. The role of the precuneus and posterior cingulate cortex in the neural routes to action. *Computer Assisted Surgery* 24:113–120. DOI: 10.1080/24699322.2018.156009.

Whatmough C, Chertkow H, Murtha S, Hanratty K. 2002. Dissociable brain regions process object meaning and object structure during picture naming. *Neuropsychologia* 40:174–186. DOI: 10.1016/S0028-3932(01)00083-5.

Wu HC, Nagasawa T, Brown EC, Juhasz C, Rothermel R, Hoechstetter K, Shah A, Mittal S, Fuerst D, Sood S. 2011. Gamma-oscillations modulated by picture naming and word reading: Intracranial recording in epileptic patients. *Clinical Neurophysiology* 122:1929–1942. DOI: 10.1016/j.clinph.2011.03.011.

Yang J, Ye J, Wang R, Zhou K, Wu YJ. 2018. Bilingual contexts modulate the inhibitory control network. *Frontiers in Psychology* 9:395. DOI: 10.3389/fpsyg.2018.00395.

Zhang Y, Wang K, Yue C, Mo N, Wu D, Wen X, Qiu J. 2018. The motor features of action verbs: fMRI evidence using picture naming. *Brain and Language* 179:22–32. DOI: 10.1016/j.bandl.2018.02.002.
