# Supplementary figures and images for: Brain hothubs and dark functional networks: correlation analysis between amplitude and connectivity for Broca’s aphasia
Source: PeerJ. 2020 Oct 1;8:e10057. doi: 10.7717/peerj.10057 (PMC7533062; doi:10.7717/peerj.10057)

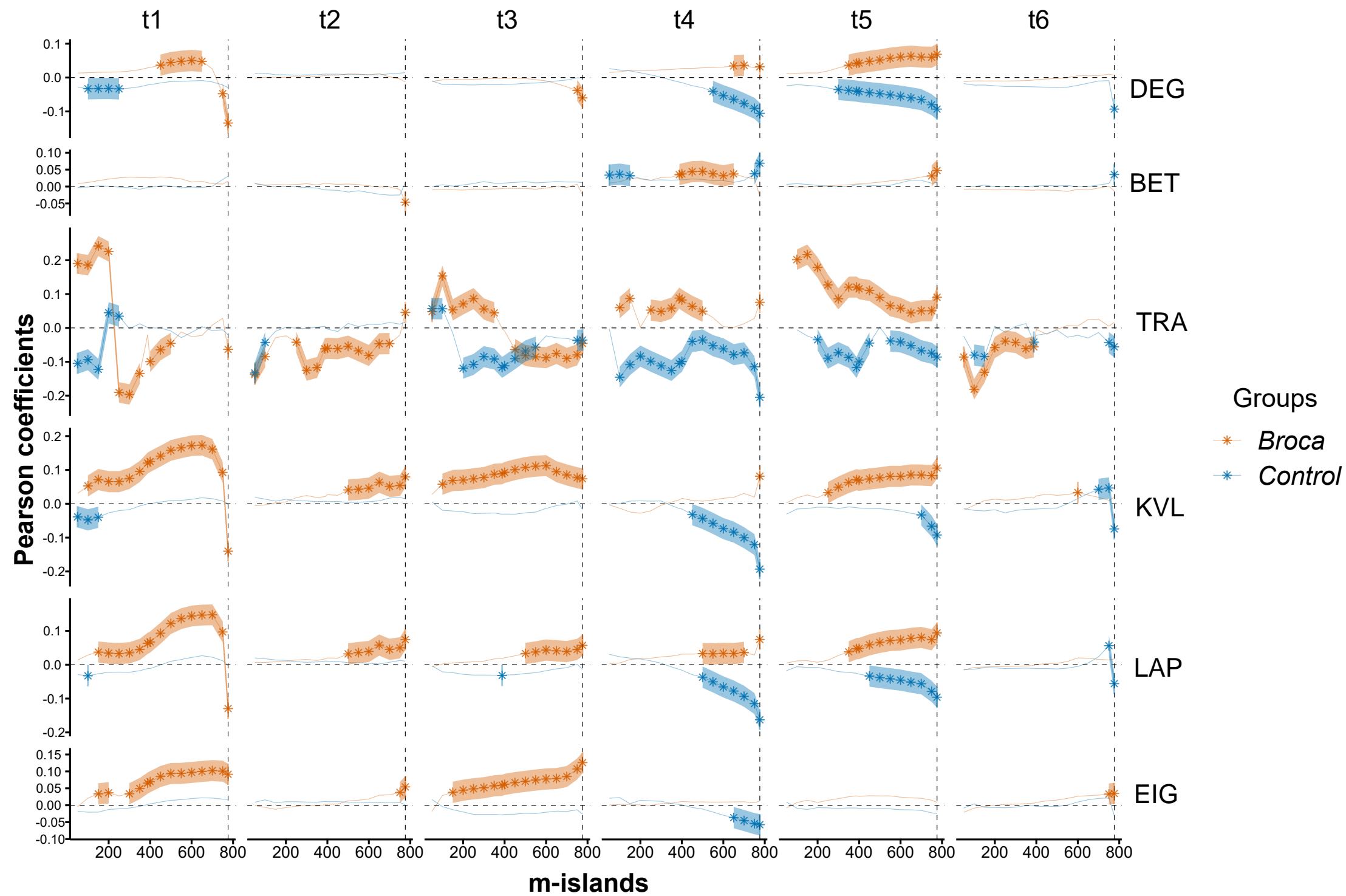

Supplement: Figure S1 — Activations are the estimated electric densities (in a physical unit of picoampere) in the source space. The partial Pearson coefficients between the activations and graph measures are calculated for a series of m-islands at different stages. The vertical dashed lines denote the maximum island size of 776. The significant coefficients (p¡0.05) are marked with asterisks. The 95% confidence intervals having significant coefficients are marked by transparent colored ribbons. DEG: weighted degree; BET: weighted betweenness; TRA: weighted transitivity; KVL: k-value of coreness; LAP: Laplacian centrality; EIG: eigenvector centrality; t1: 0–119 ms, visual feature extraction; t2: 120–150 ms, object recognition; t3: 151–190 ms, memory access; t4: 191–320 ms, semantic processing; t5: 321–480 ms, phonological encoding; and t6: 481–535 ms, articulation. A positive coefficient marked with an asterisk denotes that strongly activated brain regions are more likely to be highly connected hubs. A negative coefficient marked with an asterisk suggests that highly connected hubs are more likely to be with weak intensities of activation. The separation of the confidence intervals with opposite values of coefficients infers that the two groups have significantly different amplitude–connectivity relationships. One significant correlation with another nonsignificant correlation also implies that there are interconditional differences of amplitude–connectivity relationships. Larger m values of islands imply that the weak connections remain in networks after thresholding operations and that the islands are dense with many of weakly weighted edges. Small m values of islands imply that the weak connections are trimmed out from the networks by thresholding operations and that the islands are sparse with remaining strongly weighted edges. [file peerj-08-10057-s004.pdf]

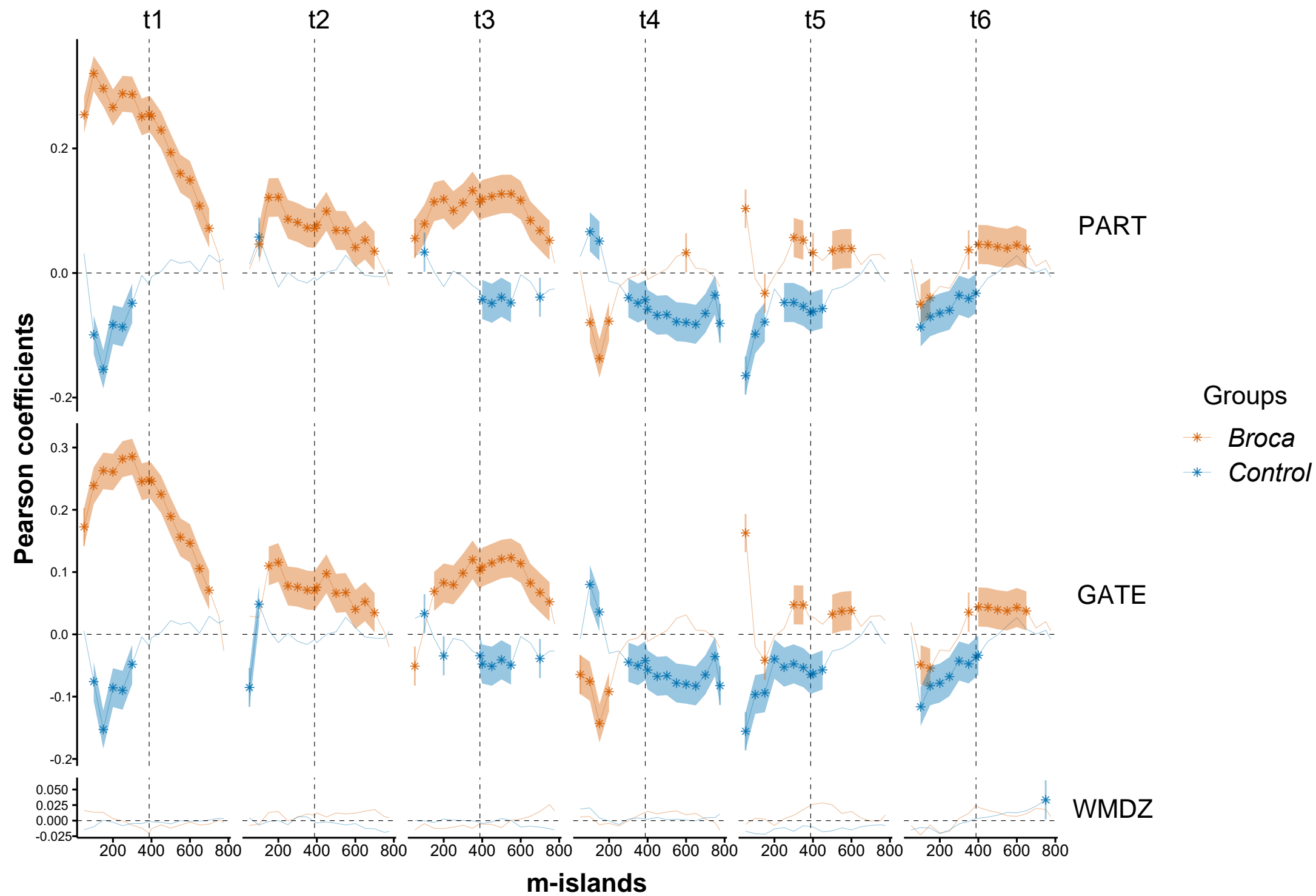

Supplement: Figure S2 — Activations are the estimated electric densities (in a physical unit of picoampere) in the source space. The partial Pearson coefficients between the activations and graph measures are calculated for a series of m-island networks at different stages. The vertical dashed lines denote the maximum island size of 388 (i.e., the number of vertices in one hemisphere). The significant coefficients (p¡0.05) are marked with asterisks. The 95% confidence intervals with significant coefficients are marked by transparent colored ribbons. PART: participation coefficient; GATE: gateway coefficient; WMDZ: within module degree z-score; t1: 0–119 ms, visual feature extraction; t2: 120–150 ms, object recognition; t3: 151–190 ms, memory access; t4: 191–320 ms, semantic processing; t5: 321–480 ms, phonological encoding; and t6: 481–535 ms, articulation. A positive coefficient marked with an asterisk denotes that strongly activated brain regions are more likely to be highly connected hubs. A negative coefficient marked with an asterisk suggests that highly connected hubs are more likely to have weak intensities of activation. The separation of the confidence intervals with opposite values of coefficients infers that the two groups have significantly different amplitude–connectivity relationships. One significant correlation with another nonsignificant correlation also implies that there are interconditional differences of amplitude–connectivity relationships. Larger m values of islands imply that the weak connections remain in networks after thresholding operations and that the islands are dense with many of weakly weighted edges. Small m values of islands imply that the weak connections are trimmed out from the networks by thresholding operations and that the islands are sparse with remaining strongly weighted edges. [file peerj-08-10057-s005.pdf]

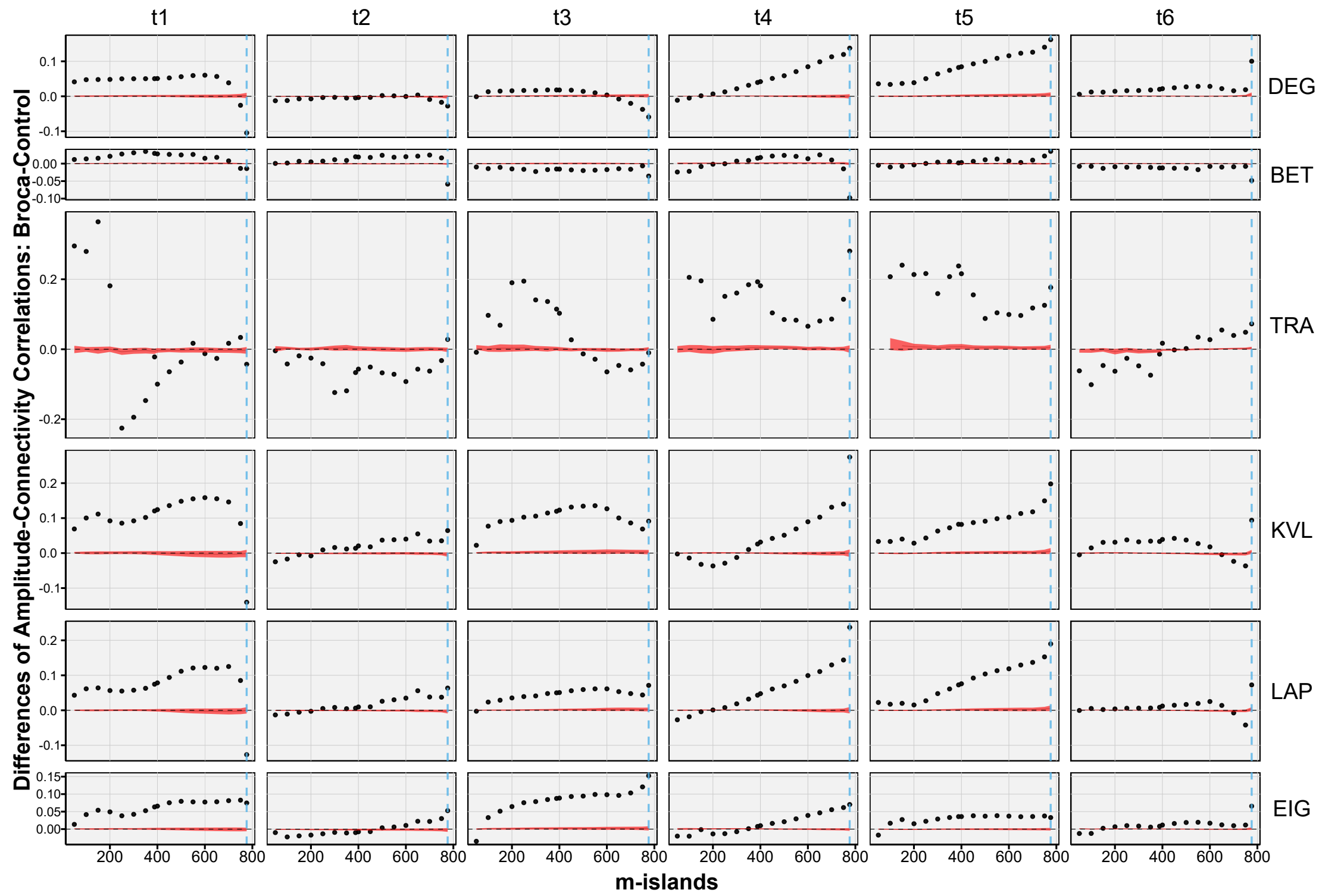

Supplement: Figure S3 — This figure shows the results of the permutation test for the module-independent graph measures. Black dots denote observed values of differences of amplitude–connectivity partial correlation Pearson coefficients between the Broca group and the Control group. Red ribbons denote the 95% confidence intervals of 1,000 random permutations. If an observed value of the difference (black dot) is not in the 95% confidence interval (red ribbon) of the random permutation, the observed difference is significant (viz., the partial Pearson coefficients are significantly different between the two groups). The vertical dashed lines denote the maximum island size of 776. DEG: weighted degree; BET: weighted betweenness; TRA: weighted transitivity; KVL: k-value of coreness; LAP: Laplacian centrality; EIG: eigenvector centrality; t1: 0–119 ms, visual feature extraction; t2: 120–150 ms, object recognition; t3: 151–190 ms, memory access; t4: 191–320 ms, semantic processing; t5: 321–480 ms, phonological encoding; and t6: 481–535 ms, articulation. [file peerj-08-10057-s006.pdf]

# Permutation Test on Inter-group Differences of Correlation Coefficients

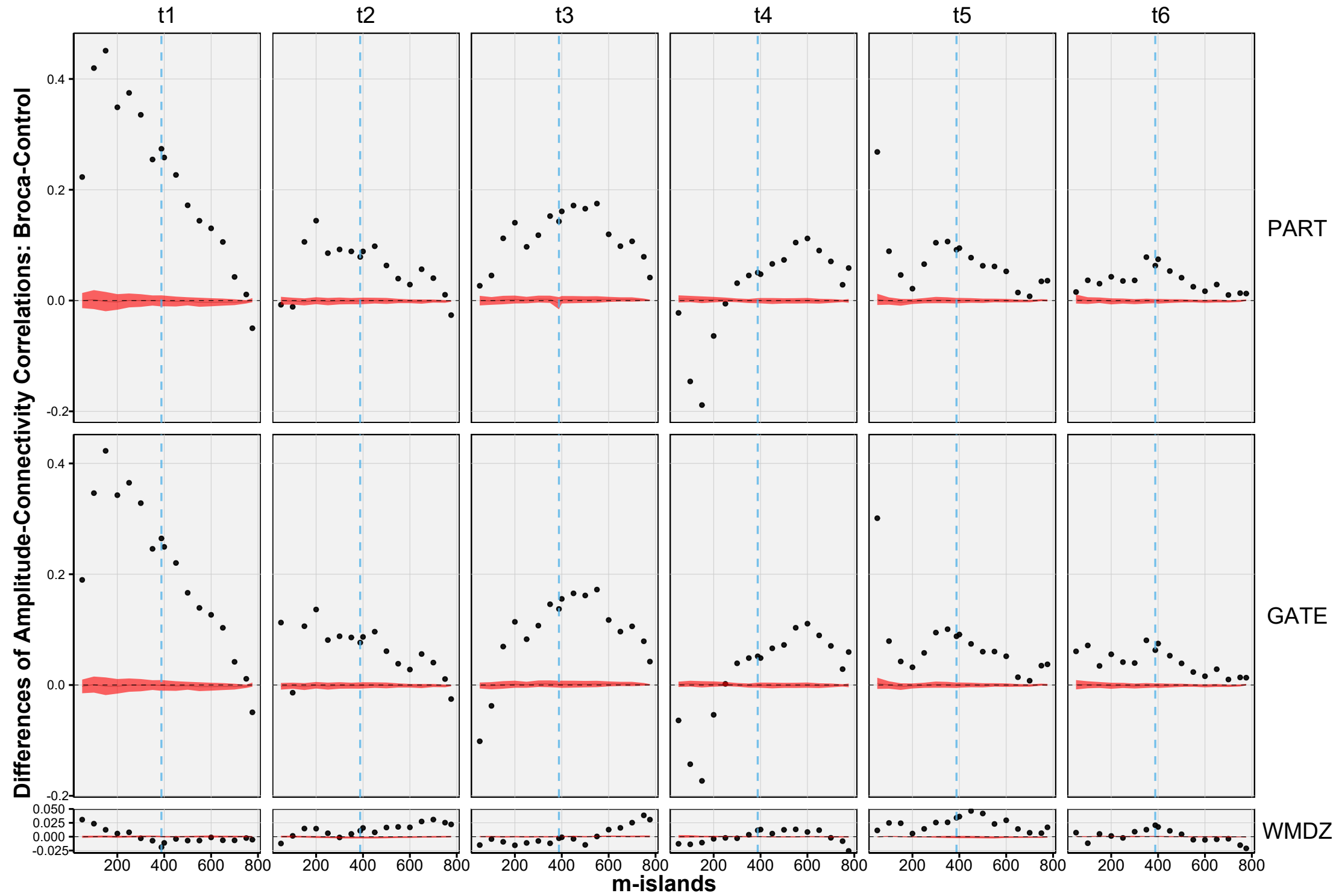

Supplement: Figure S4 — This figure shows the results of the permutation test for the module-dependent graph measures. Black dots denoted observed values of differences of amplitude–connectivity partial correlation Pearson coefficients between the Broca group and the Control group. Red ribbons denoted the 95% confidence intervals of 1,000 random permutations. If an observed value of the difference (black dot) is not in the 95% confidence interval (red ribbon) of the random permutation, the observed difference is significant (viz., the partial Pearson coefficients are significantly different between the two groups). The vertical dashed lines denote the maximum island size of 388. PART: participation coefficient; GATE: gateway coefficient; WMDZ: within module degree z-score; t1: 0–119 ms, visual feature extraction; t2: 120–150 ms, object recognition; t3: 151–190 ms, memory access; t4: 191–320 ms, semantic processing; t5: 321–480 ms, phonological encoding; and t6: 481–535 ms, articulation. [file peerj-08-10057-s007.pdf]

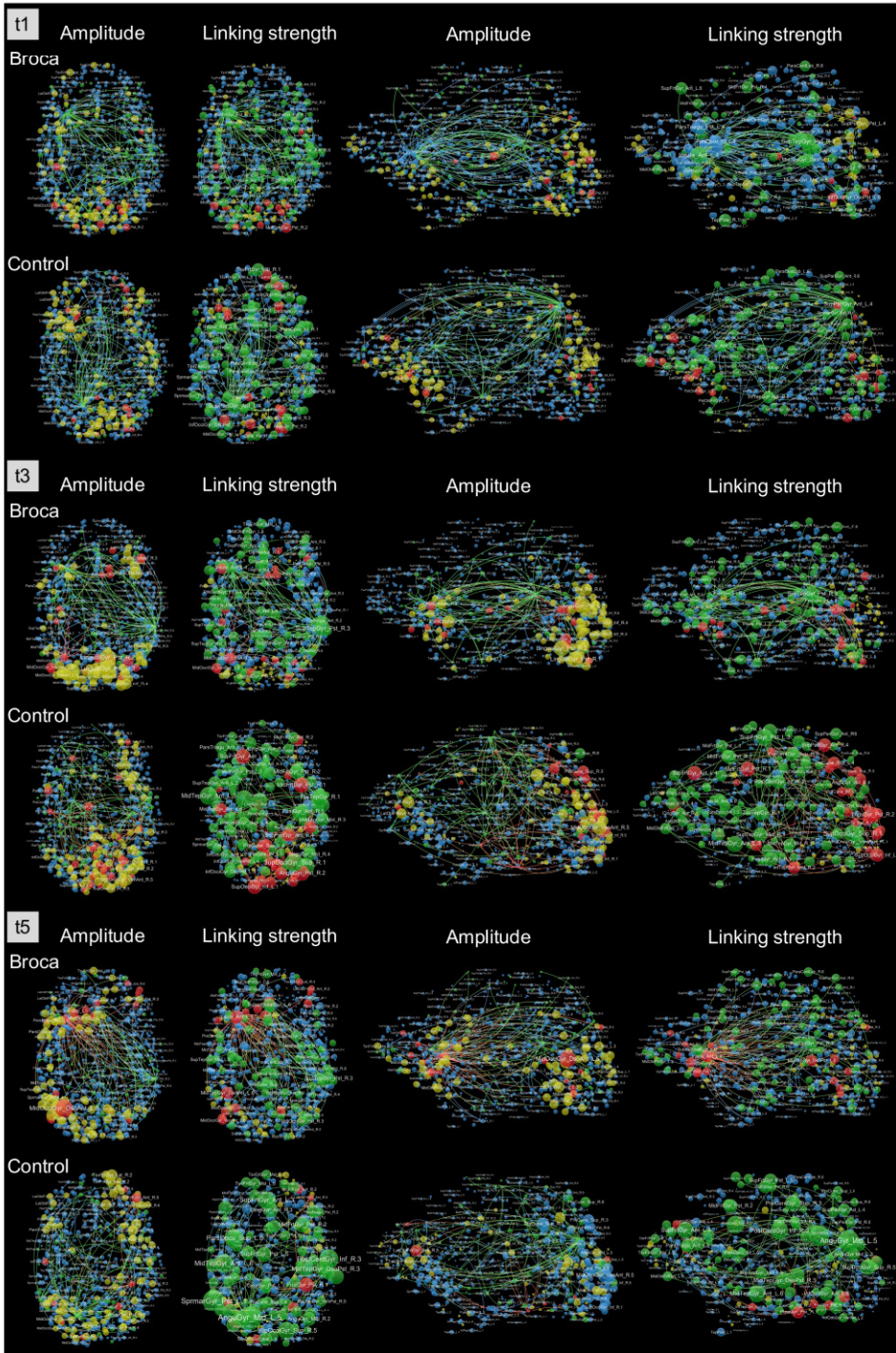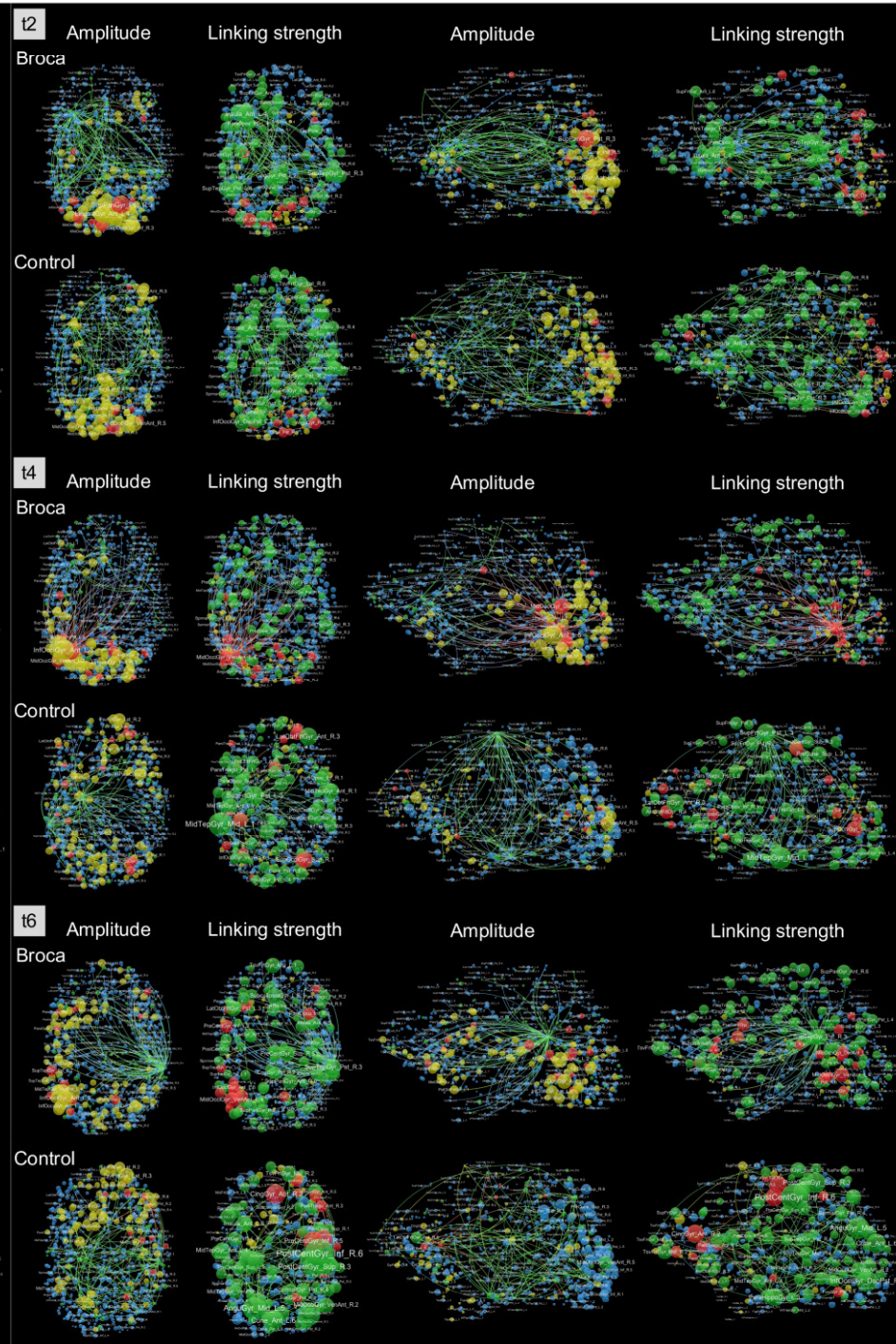

Supplement: Figure S5 — Hotspots are identified based on their amplitudes, which exceeded the mean plus one standard deviation. Hubs are identified based on their eigenvector centralities, as defined in Pajek, with the same number of hotspots at each stage. For each viewpoint, node areas on the left panel are in proportion to the z-scores of their amplitudes, whereas those on the right panel are in proportion to the total linking strengths (i.e., weighted degrees). Arrow a: Left SupFrtGyr_Pst (left superior frontal gyrus posterior). Arrow b: Left MidTempGyr_Mid (left middle temporal gyrus middle). The top-weighted 100 edges are plotted, and the edges are colored according to their terminals. There are four node types: hothubs (red), coldhubs (green), non-hub hotspots (yellow), and non-hub coldspots (blue). There are six stages: t1: 0–119 ms, visual feature extraction; t2: 120–150 ms, object recognition; t3: 151–190 ms, memory access; t4: 191–320 ms, semantic processing; t5: 321–480 ms, phonological encoding; and t6: 481–535 ms, articulation. The amplitude weighted layouts (left panels in each viewpoint) are remarkably different from the linking-strength weighted layouts (right panels in each viewpoint), suggesting that it is necessary to reconsider the role of coldhubs in functioning brain. [file peerj-08-10057-s008.pdf]

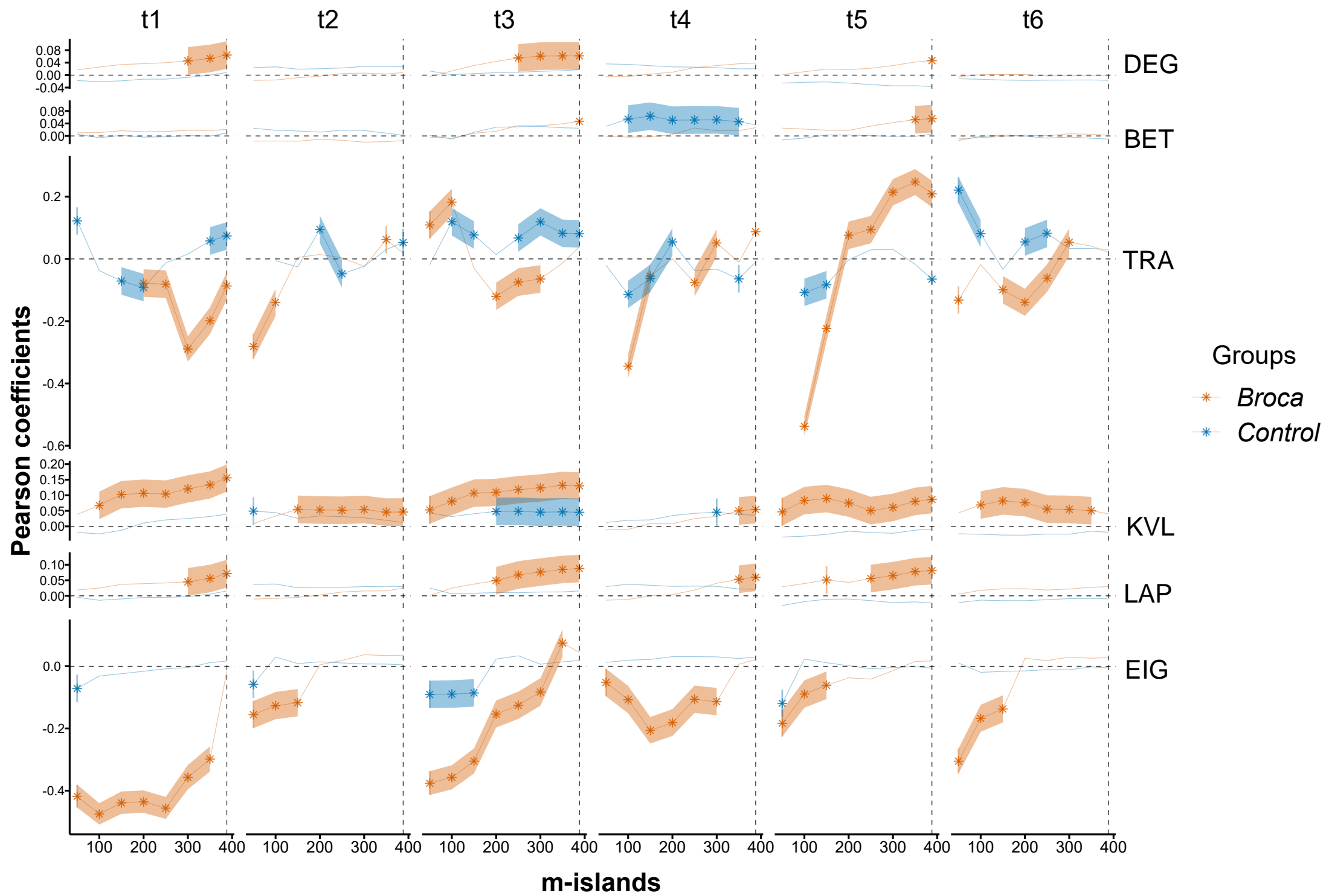

Supplement: Figure S6 — This figure shows the results of the right hemisphere (i.e., the subnetworks of the left hemisphere are deleted in the analyses). Activations are the estimated electric densities (in a physical unit of picoampere) in the source space. The partial Pearson coefficients between the activations and graph measures are calculated for a series of m-islands at different stages. The vertical dashed lines denote the maximum island size of 388. The significant coefficients (p¡0.05) are marked with asterisks. The 95% confidence intervals having significant coefficients are marked by transparent colored ribbons. DEG: weighted degree; BET: weighted betweenness; TRA: weighted transitivity; KVL: k-value of coreness; LAP: Laplacian centrality; EIG: eigenvector centrality; t1: 0–119 ms, visual feature extraction; t2: 120–150 ms, object recognition; t3: 151–190 ms, memory access; t4: 191–320 ms, semantic processing; t5: 321–480 ms, phonological encoding; and t6: 481–535 ms, articulation. A positive coefficient marked with an asterisk denotes that strongly activated brain regions are more likely to be highly connected hubs. A negative coefficient marked with an asterisk suggests that highly connected hubs are more likely to have weak intensities of activation. The separation of the confidence intervals having opposite values of coefficients infers that the two groups have significantly different amplitude–connectivity relationships. One significant correlation with another nonsignificant correlation also implies that there are interconditional differences of amplitude–connectivity relationships. Larger m values of islands imply that the weak connections remain in networks after thresholding operations and that the islands are dense with many of weakly weighted edges. Small m values of islands imply that the weak connections are trimmed out from the networks by thresholding operations and that the islands are sparse with remaining strongly weighted edges. [file peerj-08-10057-s009.pdf]

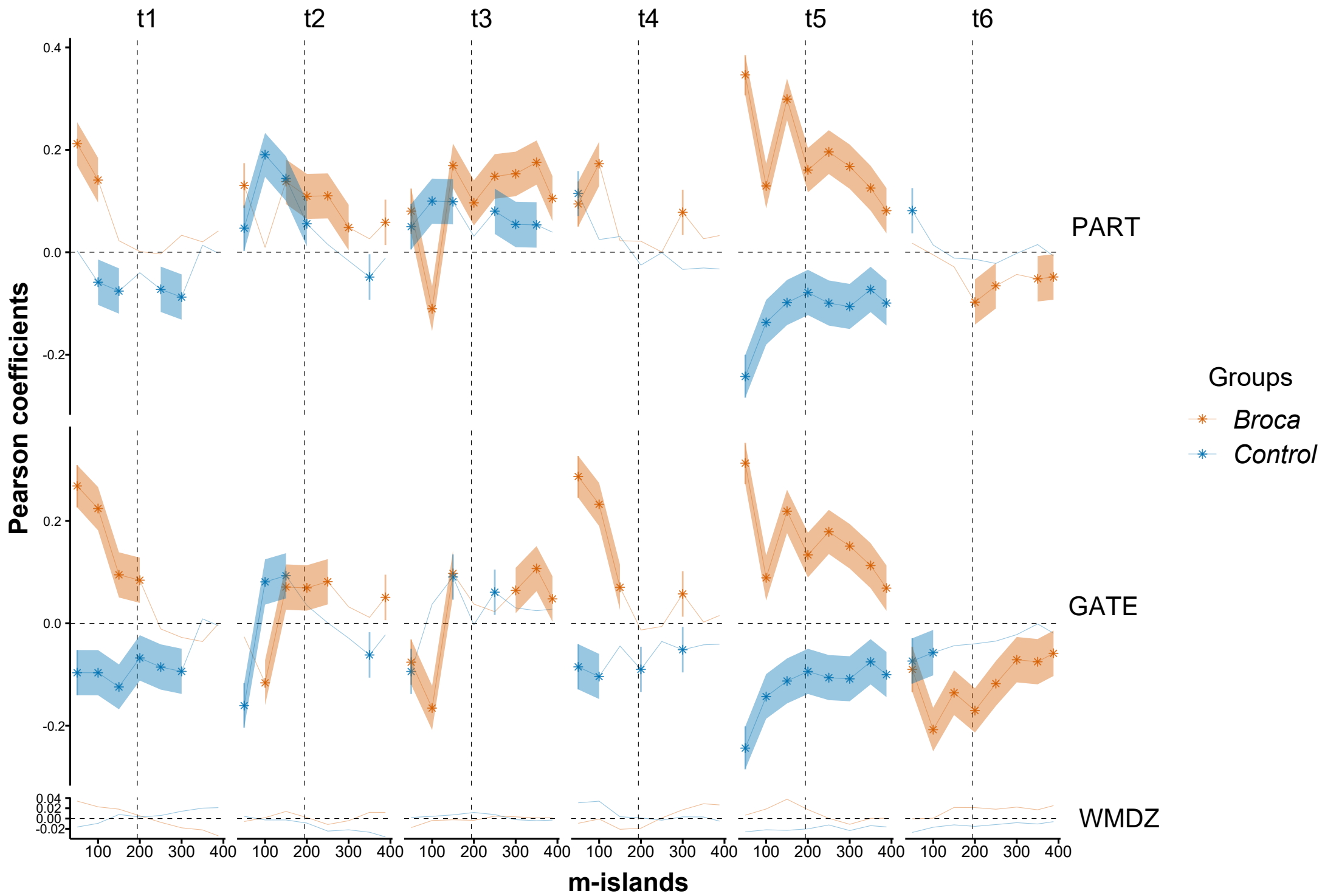

Supplement: Figure S7 — This figure shows the results of the right hemisphere (i.e., the subnetworks of the left hemisphere are deleted in the analyses). Activations are the estimated electric densities (in a physical unit of picoampere) in the source space. The partial Pearson coefficients between the activations and graph measures are calculated for a series of m-island networks at different stages. The vertical dashed lines denote the maximum island size of 199. The significant coefficients (p¡0.05) are marked with asterisks. The 95% confidence intervals with significant coefficients are marked by transparent colored ribbons. PART: participation coefficient; GATE: gateway coefficient; WMDZ: within module degree z-score; t1: 0–119 ms, visual feature extraction; t2: 120–150 ms, object recognition; t3: 151–190 ms, memory access; t4: 191–320 ms, semantic processing; t5: 321–480 ms, phonological encoding; and t6: 481–535 ms, articulation. A positive coefficient marked with an asterisk denotes that strongly activated brain regions are more likely to be highly connected hubs. A negative coefficient marked with an asterisk suggests that highly connected hubs are more likely to have weak intensities of activation. The separation of the confidence intervals having opposite values of coefficients infers that the two groups have significantly different amplitude–connectivity relationships. One significant correlation with another nonsignificant correlation also implies that there are interconditional differences of amplitude–connectivity relationships. Larger m values of islands imply that the weak connections remain in networks after thresholding operations and that the islands are dense with many of weakly weighted edges. Small m values of islands imply that the weak connections are trimmed out from the networks by thresholding operations and that the islands are sparse with remaining strongly weighted edges. [file peerj-08-10057-s010.pdf]
